# Hybrids of Fullerenes and 2D Nanomaterials

**DOI:** 10.1002/advs.201800941

**Published:** 2018-09-02

**Authors:** Muqing Chen, Runnan Guan, Shangfeng Yang

**Affiliations:** ^1^ Hefei National Laboratory for Physical Sciences at Microscale CAS Key Laboratory of Materials for Energy Conversion Department of Materials Science and Engineering Synergetic Innovation Center of Quantum Information and Quantum Physics University of Science and Technology of China Hefei 230026 China

**Keywords:** 2D nanomaterials, black phosphorus, boron nitride, fullerene, graphene, graphitic carbon nitride, transition‐metal dichalcogenide

## Abstract

Fullerene has a definite 0D closed‐cage molecular structure composed of merely sp^2^‐hybridized carbon atoms, enabling it to serve as an important building block that is useful for constructing supramolecular assemblies and micro/nanofunctional materials. Conversely, graphene has a 2D layered structure, possessing an exceptionally large specific surface area and high carrier mobility. Likewise, other emerging graphene‐analogous 2D nanomaterials, such as graphitic carbon nitride (g‐C_3_N_4_), transition‐metal dichalcogenides (TMDs), hexagonal boron nitride (h‐BN), and black phosphorus (BP), show unique electronic, physical, and chemical properties, which, however, exist only in the form of a monolayer and are typically anisotropic, limiting their applications. Upon hybridization with fullerenes, noncovalently or covalently, the physical/chemical properties of 2D nanomaterials can be tailored and, in most cases, improved, significantly extending their functionalities and applications. Here, an exhaustive review of all types of hybrids of fullerenes and 2D nanomaterials, such as graphene, g‐C_3_N_4_, TMDs, h‐BN, and BP, including their preparations, structures, properties, and applications, is presented. Finally, the prospects of fullerene‐2D nanomaterial hybrids, especially the opportunity of creating unknown functional materials by means of hybridization, are envisioned.

## 1 Introduction

Fullerene is the first member of the nanocarbon family featuring a closed‐cage structure, and the discovery of C_60_ as the first fullerene by Kroto et al. in 1985 disclosed a new era of nanoscience.^1^, ^2^ Among all known allotropes of elemental carbon, fullerene is the only soluble species with a definite spherical molecular structure merely composed of sp^2^‐hybridized carbon atoms.^3^ The unique 0D structures of fullerenes enable their use as important building blocks to construct supramolecular assemblies and micro/nanofunctional materials applicable in optoelectronic devices, catalysis, biomedicines, and so on.^4^, ^5^, ^6^, ^7^, ^8^ In particular, C_60_ has a triply degenerate low‐lying lowest unoccupied molecular orbital (LUMO), rendering an excellent electron‐accepting ability for holding up to six electrons and facilitating the formation of donor–acceptor dyads.^6^ Furthermore, fullerene can be regarded as an electron‐deficient polyalkene, and thus, it is chemically reactive.^9^ This makes the derivatization of fullerenes feasible so as to extend their functionalities.

Unlike fullerene with a 0D closed‐cage molecular structure, graphene has a 2D layered structure, offering an exceptionally large specific surface area (2630 m^2^ g^−1^), high carrier mobility (≈15 000 cm^2^ V^−1^ s^−1^ at room temperature), good optical transparency (≈97.7%), high Young's modulus (≈1 Tpa), and excellent thermal conductivity (3000–5000 W m^−1^ K^−1^).^10^, ^11^, ^12^ Hence, graphenes have attracted widespread interest and been applied in versatile fields, including sensing, field‐effect transistors (FETs), energy conversion and storage, catalysis, etc.^13^, ^14^, ^15^ However, note that such peculiar properties of graphene exist only in the monolayer and are typically anisotropic, limiting the material's applications.^16^ Therefore, considerable efforts have been made on developing different exfoliation methods of graphene and hybridizing graphene with other functional materials so as to extend the applications of graphene. For the latter approach, both noncovalent and covalent hybridizations of graphene have been implemented, and particularly, fullerenes were involved in both cases.^17^, ^18^, ^19^, ^20^, ^21^ In terms of the noncovalent hybridizations of graphene and fullerene, depositing fullerene onto a graphene layer to form a graphene/fullerene bilayer film is simple and provides a model system to investigate the charge transfer between graphene and fullerene.^22^, ^23^, ^24^, ^25^, ^26^, ^27^ Additionally, physically blending pristine fullerene (or a fullerene derivative) and graphene appears effective in constructing fullerene–graphene hybrids.^28^, ^29^, ^30^, ^31^ On the other hand, the covalent functionalization of graphene can impose more significant changes on its electronic and band structures; while constructing covalent hybrids of graphene with fullerene is more challenging, and the divergence between the chemical reactivities of graphene and fullerene should be taken into consideration, so far, only a few graphene–fullerene covalent hybrids have been successfully synthesized via esterification, coupling, cycloaddition reactions, etc.^32^, ^33^, ^34^, ^35^, ^36^ Although both covalent and noncovalent functionalizations of graphene have been extensively reviewed by several groups, few Reviews have specifically focused on the hybrids of graphene and fullerenes.

During the last decade, several other types of emerging graphene‐analogous 2D nanomaterials have been discovered.^37^, ^38^, ^39^ Among them, graphitic carbon nitride (g‐C_3_N_4_), as the most stable allotrope of carbon nitrides under ambient conditions, has a unique structure comprised of a 2D sheet of tri‐s‐triazine connected via tertiary amines. g‐C_3_N_4_ has a high thermal stability up to ≈600 °C and is chemically inert toward acids, bases, and organic solvents. In particular, g‐C_3_N_4_, with a moderate band gap and indirect‐semiconductor nature, has become a promising metal‐free catalyst for water splitting and the degradation of pollutants due to the advantages of low cost and high specific surface area (2500 m^2^ g^−1^ for the monolayer) and has been applied in organic solar cells.^40^, ^41^, ^42^, ^43^, ^44^ However, with a relatively large band gap (≈2.7 eV) and the existence of contact resistance between the nanosheets, g‐C_3_N_4_ exhibits a poor electrical conductivity and low photocatalytic activity owing to the rapid recombination of photogenerated electron–hole pairs. Thus, improvements in the photocatalytic activity of g‐C_3_N_4_ to extend this material to further applications are highly desired. To this end, the hybridization of g‐C_3_N_4_ with other functional materials, including fullerenes, has been developed in recent years and appears feasible since the polymeric nature of g‐C_3_N_4_ renders the chemical structure flexible.^45^, ^46^, ^47^, ^48^, ^49^, ^50^, ^51^, ^52^, ^53^, ^54^, ^55^

The inorganic transition‐metal dichalcogenides (TMDs) represent another type of emerging 2D semiconducting nanomaterial and have been attracting widespread attention due to their intriguing electronic, optical, and mechanical properties.^56^, ^57^, ^58^, ^59^, ^60^ MoS_2_, consisting of hexagonal rings with Mo and S atoms alternately located at the hexagon corners, is the most representative TMD with a direct band gap in monolayer form and high in‐plane carrier mobility, thus being suitable for versatile applications in electro and photocatalysis, photovoltaics, and photoelectric devices.^61^, ^62^, ^63^ For the applications of MoS_2_ in photocatalytic H_2_ production, since only its edges have a high catalytic activity, whereas its basal plane is inactive, a low photocatalytic activity has been achieved, even for single‐layer MoS_2_. Consequently, MoS_2_ is generally applied as a cocatalyst loaded on conventional metal‐based semiconductor photocatalysts, such as TiO_2_, CdS, and ZnO, to reduce the energy barrier of water splitting and to increase the stability of the photocatalysts. To enhance the photocatalytic H_2_ production activity of MoS_2_ itself, the construction of nanocomposites composed of MoS_2_ and other functional materials, such as graphenes and fullerenes, provides a practical solution.^64^, ^65^, ^66^, ^67^ Similar concepts have been applied to construct hybrids of fullerene and other 2D nanomaterials, including hexagonal boron nitride (h‐BN) and black phosphorus (BP), though only a few reports are available, and charge transfer between fullerene and the 2D nanomaterials may occur, leading to certain performance improvements.^68^, ^69^, ^70^, ^71^, ^72^ For these graphene‐analogous 2D nanomaterials, especially g‐C_3_N_4_ and the TMD MoS_2_, no Review has reported their hybridization with fullerenes, which is crucial for understanding such intriguing 0D–2D hybrid systems.

In this Review, we focus on the hybrids of 0D fullerenes and 2D nanomaterials, including their preparations, structures, properties, and applications. Based on known 2D nanomaterials, such as graphene, g‐C_3_N_4_, TMDs, h‐BN, and BP, all types of hybrids of fullerenes and 2D nanomaterials reported to date are exhaustively reviewed, with emphasis on the exclusive impact of fullerenes with unique electronic and chemical properties on the electronic and band structures of 2D nanomaterials. Upon attachment of fullerene molecules, noncovalently or covalently, the physical/chemical properties of 2D nanomaterials can be tailored, and in most cases, improved, and the mechanisms responsible for such improvements are discussed in detail. Furthermore, fullerene incorporation may induce new properties in 2D nanomaterials as a result of strong intermolecular interactions, thus extending the functionalities and applications of 2D nanomaterials. At the end, we envision the prospects of fullerene‐2D nanomaterial hybrids, especially the opportunity of creating unknown functional materials by means of hybridization.

## 2 Hybrids of Fullerenes and Graphenes

### 2.1 Noncovalent Hybrids of Fullerenes and Graphenes

Noncovalent hybridizations of fullerenes and graphenes have been fulfilled by depositing fullerene onto a graphene layer to form a graphene/fullerene bilayer film and by physically blending pristine fullerenes or fullerene derivatives and graphenes (see **Table** **1**).

**Table 1:** Noncovalent hybrids of fullerenes and graphenes

Type	Raw material		Preparation method	Application/performance	Ref.
	Fullerene	Graphene			
Bilayer films	Pristine	Pristinea)	Thermal evaporation deposition	Excited‐state charge transfer	73
	Pristine	Pristine	Thermal evaporation deposition	Graphene Moiré pattern	23
	Pristine	Pristine	chemical vapor deposition	Negative photoconductivity	26
	Pristine	Pristine	Spray coating	FET/p‐type behavior	74
	Pristine	Pristine	Thermal evaporation deposition	FET/On/off ratio: 3 × 10^3^	25
	Pristine	Pristine	Thermal evaporation deposition	Strain lattice imprinting	24
	Pristine	Pristine	Thermal evaporation deposition	–	20
	Pristine	Pristine	–	Single‐molecule junctions	22
	Derivative	Pristine	Thermal evaporation deposition	Single‐molecule junctions	75
	Derivative	Pristine	Immersed method	Single‐molecule junctions	76
	Derivative	Pristine	Immersed method	Single‐molecule junctions	77
Physical blends	Pristine	Pristine	Acid treatment	Lithium ion batteries/capacity: 784 mAh g^−1^	78
	Pristine	GOb)	Thermal treatment	Supercapacitors/capacity: 135.36 F g^−1^	31
	Pristine	rGOc)	Liquid‐liquid interfacial precipitaion	FET devices/p‐type behavior	30
	Pristine	Pristine	Ultrasonic treatment	Solar cell PCE: 0.85%	83
	Pristine	GO	Ultrasonic treatment	Catalysis/redox of biomolecules	84
	Pristine	GO	Ultrasonic treatment	Sensor/oxidation of cis‐jasmone	85
	Pristine	rGOc)	Ultrasonic treatment	Sensor/detection of glucose	87
	Pristine	GO	Ultrasonic treatment	Sensor/detection of dopamine	88
	Pristine	GO	Langmuir–Schaefer deposition technique	Electrical conductivity	89
	Derivative	Pristine	Deposition grown	Memory effects	29
	Derivative	rGOc)	Immersed method	Polymer solar cells/PCE: 3.89%	28
	Derivative	Pristine	Ultrasonic treatment	–	90
	Derivative	GO	Ultrasonic treatment	Thermal and mechanical properties	91
	Endohedral (Sc_3_N@C_80_)	Pristine	–	–	94

^a)^ Pristine graphene was synthesized by either chemical vapor deposition (CVD) or exfoliation of graphite

^b)^ Graphene oxide (GO) was prepared by a modified Hummers method

^c)^ Reduced graphene oxide (rGO) was synthesized via reduction of GO by a reductant such as hydrazine.

#### 2.1.1 Graphene/Fullerene Bilayer Films

**Figure 1:**
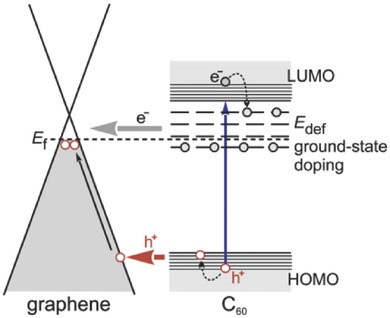
Electronic states in C_60_/graphene and photoexcited charge transfer processes. Reproduced with permission.^73^ Copyright 2015, American Chemical Society.

A simple way to construct hybrid nanostructures comprised of fullerene and graphene is to deposit C_60_ onto a graphene film to form a bilayer hybrid; in this way, the interfacial electronic interactions and charge transfer between C_60_ and graphene can be investigated. In 2015, Heinz et al. fabricated a C_60_/graphene hybrid by depositing C_60_ onto single‐layer graphene and studied the ground and excited‐state charge transfers at the C_60_/graphene interface by using Raman spectroscopy and THz time‐domain spectroscopy. The authors concluded that at equilibrium the C_60_ layer functioned as an electron acceptor affording hole doping of the graphene with an injection of ≈0.04 holes per interfacial C_60_ molecule at the surface of the bilayer hybrid, as well as a downshift of the graphene Fermi level by 160 meV (**Figure** **1**).^73^ Furthermore, such hole doping was accompanied by an increase in the graphene carrier mobility and a charge transfer process from C_60_ molecules near the interface with an injection efficiency of ≈0.3.^73^

**Figure 2:**
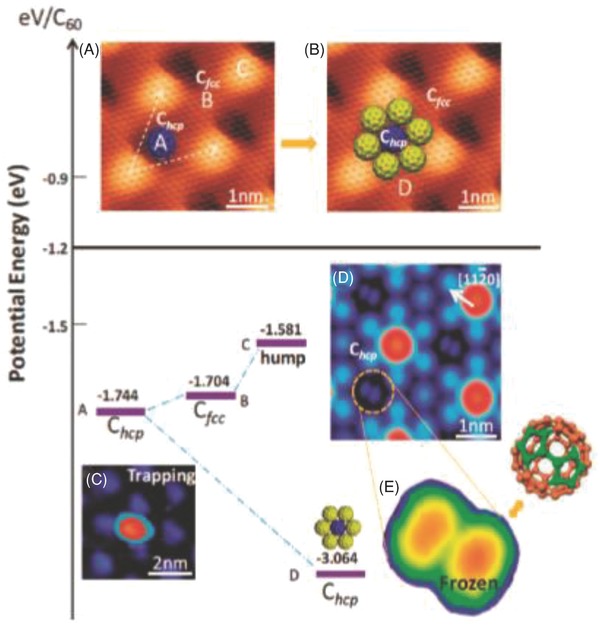
Hierarchical adsorption energies of C_60_ molecules in different sites of the graphene Moiré pattern. A) Scheme of an individual C_60_ molecule preferentially trapped in the C_hcp_ valley at room temperature (RT) and its corresponding STM image as shown in (C). B) Scheme of six C_60_ molecules attached to the trapped C_60_ as a seed for the nucleation of monolayer C_60_ islands; C_60_–C_60_ cohesive energy increases. D) RT freezing of the thermal motions of C_60_ in the Chcp valleys once a C_60_ monolayer is formed. E) All C_60_ molecules trapped in C_hcp_ valleys display a dumbbell shape, aligning along the 〈11–20〉 directions. The bright lobes in the dumbbell‐shaped correspond to pentagons of the C_60_ cage at positive sample bias, which suggests C_60_ orients with the 6:6 bond (the C—C bond between two carbon hexagons) facing upward, as shown in the right top of (E). Reproduced with permission.^23^ Copyright 2012, American Chemical Society.

In 2012, Loh and co‐workers prepared an ordered graphene Moiré pattern with a lattice constant of ≈30 Å on Ru(0001), which provided a unique surface with humps and valleys facilitating the assembly of C_60_ molecules atop (**Figure** **2**).^23^ The chemical hybridization of graphene between carbon atoms in the valley regions and Ru(0001) resulted in a back‐donated charge transfer to graphene and the electron doping of the valley region, and thus, the electron enrichment of the valley region increases its reactivity to C_60_ molecules.^23^ More importantly, due to the large trapping energy at the Moiré valleys, the molecular orientation and spatial alignment of individual C_60_ molecules can be frozen at room temperature, facilitating applications in molecular spintronics. Furthermore, C_60_ molecules underwent polymerization at elevated temperatures and acted as a solid carbon precursor source for the homoepitaxial growth of graphene nanostructures.^23^

In 2013, Qiu and co‐workers constructed a fullerene/graphene hybrid device by depositing self‐assembled C_60_ films onto graphene sample surfaces using a spray coating method and investigated the electrical interaction and charge transfer kinetics at the graphene/adsorbate interface by using flicker noise, as an insightful probe, in combination with Raman spectroscopy and DC‐transport measurements.^74^ Their results unveiled a significant variation in the noise spectra obtained on the same graphene transistors before and after C_60_ deposition, which was attributed to the carrier trapping/detrapping processes in the hybrid system, finally leading to an enhanced charge noise. Meanwhile, the charge exchange process was significantly suppressed with decreasing temperatures, indicating that the flicker noise acted as a powerful probe to elucidate the nature of the charge transfer in the graphene–fullerene hybrid device. Furthermore, the authors fabricated graphene field‐effect transistors to quantitatively evaluate the charge transfer between C_60_ molecules and graphene and found that a large number of carriers with a high Fermi energy for n‐doped graphene may overcome the energy barrier to occupy the LUMO of C_60_ molecules compared to that of p‐doped graphene.^74^The number of electrons injecting from graphene to C_60_ was estimated to be 0.6–1.3 × 10^12^ cm^−2^, ≈0.01 electrons captured by a single C_60_ molecule.^74^

Likewise, in 2014, Mendoza et al. constructed a fullerene/few‐layer graphene (FLG) bilayer system by thermally evaporating C_60_ onto FLG films prepared by a chemical vapor deposition (CVD) technique. The electrical conductance of the C_60_/FLG system decreased when the system was under illumination and was attributed to a kind of negative photoconductivity.^26^ More interestingly, when the light intensity illuminating the C_60_/FLG system varied, FLG exhibited p‐type doped characteristics under low light intensities and presented n‐type doped behavior for high light intensities.^27^

Later, in 2015, Bao et al. fabricated a C_60_–graphene vertical heterostructure with a well‐ordered film morphology on the graphene, as confirmed by transmission electron microscopy (TEM).^26^ They observed strong epitaxial relations between the C_60_ crystal and graphene lattice directions. The preferential growth of C_60_ crystals is prone to graphene's zigzag and armchair directions. Moreover, the C_60_–graphene vertical junction‐based transistors showed n‐type transport behavior. The energy barrier modulation at the C_60_–graphene Schottky junction can achieve a high on/off ratio above 3 × 10^3^, which is difficult to obtain for conventional graphene transistors. Thus, graphene is demonstrated to offer an excellent substrate for the epitaxial assembly of organic semiconductors toward electronics applications.^26^

In the same year, Reinke and co‐workers reported that the intercalation of C_60_ molecules at the graphene/Cu interface by annealing led to amorphous and crystalline structures.^24^ The intercalated C_60_ molecules imprinted a local strain/deformation on the graphene layer with various magnitudes controlled by the intermolecular distance.^24^ More importantly, a crystalline intercalated structure induces the formation of topographic as well as electronic superlattices defined by the local strain field. This work provides a new strategy for controlling local strain and, at the same time, achieving positional control of the strained regions in amorphous and crystalline structures.

**Figure 3:**
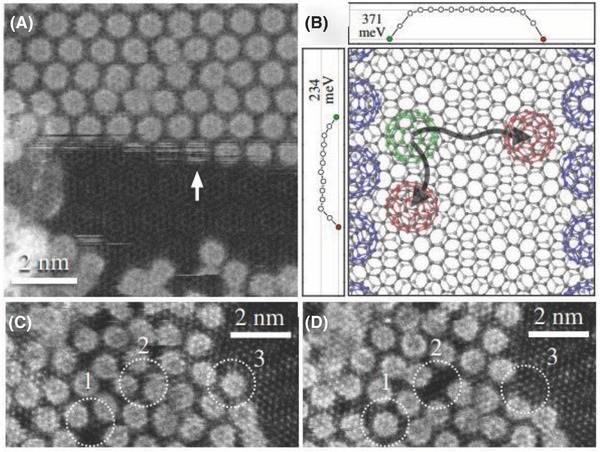
Dynamics of sandwiched fullerenes. A) An oscillating C_60_ at the edge of a gap in the fullerene monolayer, over which the graphene layers are suspended. At the location indicated by the arrow, a molecule disappears and appears at least 17 times during 100 scan lines. B) Nudged elastic band paths and energy barriers for C_60_ diffusion along an edge of fullerene monolayer (vertical) and from one edge of the gap to another (horizontal). C,D) A disordered monolayer showing a void propagating from location 1 to 2, and the C_60_ at location 3 escaping outside the field of view between the two consecutive frames. Reproduced with permission.^20^ Copyright 2017, American Association for the Advancement of Science.

Very recently, Meyer and co‐workers constructed a mixed‐dimensional sandwich heterostructure in which C_60_ molecules were encapsulated by two layers of graphene (**Figure** **3**).^20^ The fullerene monolayers in the sandwich architecture exhibited a lattice spacing of 9.6 Å, which was ≈5% smaller than the bulk spacing. Interestingly, the dynamics of entire C_60_ molecules can be clearly observed, showing that weakly bound fullerenes oscillate between different positions at the edges of 2D C_60_ molecular crystals where the graphene layers were suspended over nanometer areas, along with mobile vacancies in disordered 2D fullerene layers. Furthermore, the graphene sandwich can play the role of a nanoscale reaction chamber, providing a clean interface to the microscope vacuum and affording some suppression of radiation damage.^20^

Bilayer films comprised of graphene and fullerene derivatives have also been studied. In 2011, Lofwander and co‐workers constructed a single‐molecule device consisting of graphene and a fullerene derivative, where the graphene was patterned to form a nanogap and can be used as electrodes for gate‐tunable molecular electronics, and a dumbbell fulleropyrrolidine consisting of a central wire of 1,4‐phenylenediamine attached to C_60_ acted as a single molecule.^22^ Their results revealed that the C_60_‐on‐graphene bonding was beyond simple physisorption, as the charge rearrangement causes a work function shift. Furthermore, the considerable charge‐transfer effects in the hole‐doped leads (*W* = 4.8 eV) results in the Fermi‐level energy of the system to align inside the broadened LUMO. When the gate voltage is changed, the charge transfer to the molecule increases through the Dirac (neutrality) point of graphene to the electron‐doped side (eventually reaching *W* = 4.5 eV), resulting in a Fermi level deeper inside the LUMO.^22^

Similar single‐molecule junctions employing a fullerene derivative were later constructed by several other groups. In 2015, using a C_60_ end‐capped molecular wire comprised of two styrene units covalently connected via a [2,2′]paracyclophane moiety in the center, Weber and co‐workers fabricated graphene–fullerene–graphene junctions with ultraflat, transparent, and robust graphene nanogaps. The single‐molecule contacts were confirmed according to the measured *I–V* curves, which were similar to those obtained with the mechanically controlled break junction technique. Additionally, nonlinear *I–V* curves were obtained in a very low yield when molecules were applied at low concentration, thus ruling out the possibility of larger numbers in the junction.^75^

In 2016, Mol and co‐workers studied a similar graphene–fullerene–graphene single‐molecule transistor with a pyrene‐functionalized C_60_ bisadduct used as the junction and observed the redox‐dependent Franck–Condon blockade and avalanche transport phenomena. The C_60_ bisadduct was synthesized through a 1,3‐dipolar cycloaddition reaction between C_60_, 1‐pyrene carboxaldehyde, and *N*‐methylglycine.^76^ Based on a combined approach of transport spectroscopy, Raman spectroscopy, and density functional theory (DFT) calculations, the authors concluded that charge transport in the bisadduct was more likely to be located on the highest occupied molecular orbital (HOMO) level. The functionalization of C_60_ with pyrene anchor groups was verified to effectively modify the electron delocalization and energy levels, leading to HOMO‐dominated transport.^76^ Meanwhile, calculations showed that the modification of electron delocalization and energies via the inclusion of side groups can be used to tune the thermopower of single‐molecule junctions. In a follow‐up study by the same group, the thermoelectric effect in such single‐molecule junctions was studied.^77^ By applying an AC voltage with a modulation frequency (*f*) to the microheater and measuring the thermovoltage (*V*_th_) drop across the device at a frequency (2*f*) for different back gate voltages (*V*_g_),^77^ high power factors can be achieved approaching the theoretical limit of a thermally and lifetime‐broadened Coulomb peak by carefully tuning the transmission of a molecular junction toward sharp isolated resonance features.^77^ To achieve a high thermoelectric performance in molecular nanodevices, the authors proposed several principles including (1) the molecular energy levels need to align closely with the Fermi level of the electrodes, (2) the tunnel coupling needs to be such that the lifetime of the transmission resonance is comparable to *k*_B_ *T*_0_ at the operating temperature, and (3) the tunnel couplings to the left and right electrodes need to be equal to achieve a maximum power factor.

#### 2.1.2 Physical Blends of Pristine Fullerene and Graphene

Noncovalent hybrids of fullerenes with graphene have also been facilely prepared by physically blending both components. In 2008, Honma and co‐workers reported a graphene–fullerene physical blend by incorporating C_60_ into graphene nanosheets prepared by the exfoliation of a bulk graphite crystal and followed by a reassembling process.^78^ The results indicated that the d‐spacing of the hybrid and the corresponding charge capacities in lithium (Li)‐ion batteries had an approximately linear relationship, and the expansion in the d‐spacing of the graphene layers may cause additional sites for the accommodation of lithium ions. As a result, the specific capacity of the graphene–C_60_ physical blend increased from 540 to 784 mAh g^−1^ after the incorporation of C_60_.^78^ To understand the mechanism of Li adsorption on a graphene–C_60_ hybrid, in 2015, Lee and co‐workers carried out DFT studies and found that charge transfer from the graphene to the C_60_ took place in the graphene–C_60_ hybrid system, leaving the graphene positively charged (+0.095 e) and the C_60_ negatively charged (−0.095 e). As a result, Li ions preferentially adsorbed on the C_60_ side, particularly in the midway region between graphene and C_60_ or between two C_60_ molecules.^79^ The adsorption energy for Li atoms on the graphene–C_60_ hybrid system (−2.285 eV) was higher than that on bare graphene (−1.375 eV), indicating that the Li adsorption on the graphene–C_60_ hybrid system is more stable.

**Figure 4:**
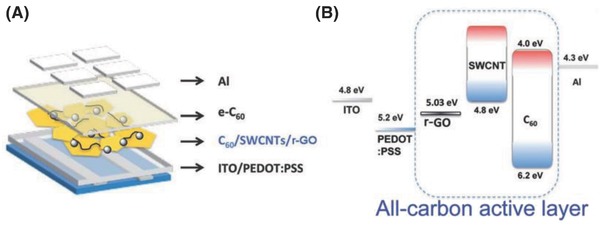
A) Schematic drawing illustrating the device configuration of a solar cell with the all‐carbon composite as the active layer. B) Corresponding schematic energy level diagram of the device. The all‐carbon active layer is highlighted within the dashed box. Reproduced with permission.^83^ Copyright 2011, American Chemical Society.

In addition to pristine graphene (pG) sheets, exfoliating graphite flakes to few‐layer graphene via chemical functionalization is currently commonly used as the most cost‐effective method of obtaining graphene due to its easiness, reliability, and scalability.^80^, ^81^ Converting graphite to graphene oxide (GO) by oxidation‐based intercalation is crucial because the interlayer van der Waals (vdW) force within graphite is decreased by the insertion of oxygen‐containing groups (epoxy, hydroxyl, and carboxyl groups) during the oxidation process.^82^ Using GO as a dispersing agent, in 2011, Huang and co‐workers developed an all‐carbon composite via the physical blending strategy, co‐assembling C_60_, pristine single walled carbon nanotubes (SWCNTs), and GO sheets.^83^ Interestingly, GO acted as a dispersing agent for all‐carbon heterojunctions, without molecular linkers or surface functionalization agents, and thin films of C_60_/SWCNTs/GO exhibited a surface roughness of only a few nanometers. After the thermal reduction of GO, the as‐prepared C_60_/SWCNTs/rGO composite was utilized as the active layer of a photovoltaic device (**Figure** **4**), affording a short‐circuit current (*J*_sc_) of 1.23 mA cm^−2^, an open‐circuit voltage (*V*_oc_) of 0.59 V, a fill factor (FF) of 0.29, and a power conversion efficiency (PCE) of 0.21%. Such a decent PCE was attributed to the presence of numerous clean p–n junctions distributed throughout the all‐carbon hybrid, facilitating the spontaneous dissociation and subsequent separation of the photogenerated excitons along the bicontinuous heterojunctions.^83^ In a follow‐up study, by replacing the C_60_ with C_70_ in both the active layer and blocking layer, a nearly fourfold increase in *J*_sc_ from 1.23 to 4.95 mA cm^−2^ was achieved, leading to a dramatic increase in PCE from 0.21% to 0.85%.^83^ The conceptually new solution‐processed all‐carbon solar cells demonstrated in these pioneering studies are promising because of the high environmental stability since all of the graphitic components are mechanically and chemically robust.

Several efforts have since been made to prepare hybrid materials based on GO or reduced graphene oxide (rGO). In 2011, Shin and co‐workers reported the preparation of hybrids of C_60_ with rGO by the liquid–liquid interfacial precipitation method, resulting in rGO‐wrapped C_60_ wires.^30^ A UV–vis spectroscopic characterization unveiled that the driving force of the assembly originated from the *π–π* interactions between rGO and C_60_. FET devices fabricated with rGO/C_60_ wires exhibited a p‐type semiconducting behavior both in vacuum and in air, indicating that the rGO, as a shell layer, can transport holes effectively. However, the pure C_60_ wires and rGO sheets showed n‐type and ambipolar behaviors, respectively, in vacuum. Furthermore, the photovoltaic devices based on rGO/C_60_ wires as the active layer exhibited a significant enhancement in short‐circuit current (*J*_SC_) from 0.07 to 0.19 mA cm^−2^ compared to the devices based on a pure rGO active layer. This result can be interpreted as the photoinduced electron transfer from rGO to C_60_ under light irradiation.^30^

**Figure 5:**
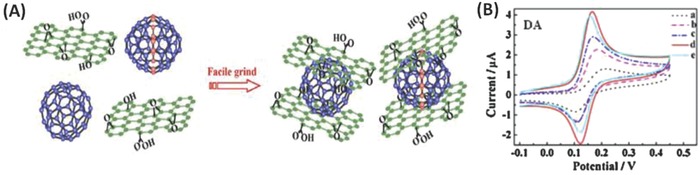
A) The scheme for noncovalent hybrid of C_60_, C_70_ with GO. B) Taking DA as an example, EIS of 5 × 10^−3^ m K_3_Fe(CN)_6_ in 0.1 m KCl at GCE (a), PTA/GCE (b), PTA–GO/GCE (c), PTA–GO–C_60_/GCE (d), and PTA–GO–C_70_/GCE (e). Reproduced with permission.^84^ Copyright 2013, Elsevier B.V.

In 2013, Hu and co‐workers prepared a noncovalent hybrid of C_60_/C_70_ with GO by a simple grinding method, and the material exhibited a solubility of 5.0 mg mL^−1^ in water when the weight ratio of the fullerenes to GO was 1:3 (**Figure** **5**A).^84^ The noncovalent interaction between the fullerenes and GO was regarded as *π–π* stacking, as verified by UV–vis spectroscopy. The fullerene/GO hybrid showed the features of a high solubility, good stability, and high electroactivity. Considering the high catalytic redox activity of phosphotungstic acid (PTA), a PTA–GO–fullerene nanofilm was further fabricated by a one‐step electrodeposition. Compared to either PTA or PTA–GO‐modified electrodes, the PTA–GO–fullerene composites showed improved catalytic activity for the oxidation of a variety of small biomolecules, including dopamine (DA), with enlarged peak currents and an apparent negative shift of peak potentials (Figure 5B). These features can be explained by the water‐soluble GO–fullerene hybrid not only increasing the surface area of the electrode but also facilitating the fixation of PTA on the electrode as conductive medium.^84^ Soon after this work, the same group extended these PTA–GO–fullerene films to the field of electrochemical sensing in terms of the direct oxidation of cis‐jasmone (CJ).^85^ The electrochemical responses of PTA–C_60_–GO/graphite electrode (GE) were found to be sensitive to the direct oxidation of CJ and provided a short analysis time, convenience, and good accuracy.^85^ The enhanced catalytic ability of PTA–C_60_–GO/GE was because the partially reduced C_60_ in the electropolymeric process exhibited a favorable conductivity as an effective electron‐transfer medium compared to that of the pure GO film.^85^

Electrochemical sensing applications of noncovalent hybrids of fullerene–graphene have also been reported by other groups. Given that the direct electron transfer properties of enzymes are helpful for studying key biological reactions and for the construction of biosensors and biofuel cells,^86^ in 2015, Lou and co‐workers prepared a noncovalent rGO–C_60_ composite by electrochemical reduction of a GO–C_60_ composite, which was then used as an immobilization matrix for GO*_x_*.^87^ The amperometric *i–t* response of an rGO–C_60_/GO*_x_*‐modified electrode to different concentrations of glucose and in constantly stirred oxygen‐saturated phosphate buffer saline (PBS) with a working potential of −0.42 V showed that the amperometric reduction current decreased with the increase in glucose concentration up to 14.6 × 10^−3^ m.^87^ The steady‐state current of the biosensor appeared at 3 s, and the amperometric *i–t* response current for the biosensor exhibited a linear pattern against the concentration of glucose in a range of 0.1–12.5 × 10^−3^ m. Hence, the rGO–C_60_/GO*_x_*‐modified electrode showed good analytical ability when used as a sensor for glucose over a wide response range with a low limit of detection, fast response time (3 s), and high sensitivity.^87^ One year later, the same group prepared a nanocomposite of C_60_‐wrapped GO by using a simple sonication method, and the material was used to modify a glassy carbon electrode.^88^ The glassy carbon electrode modified with the GO–C_60_ hybrid showed a good sensitivity with a lower oxidation overpotential toward DA than did those of the pristine GO and C_60_. The as‐prepared sensor for DA detection exhibited a linear response in a range of 0.02–73.5 × 10^−6^ m and a detection limit of 0.008 × 10^−6^ m (based on 3σ) with a sensitivity of 4.23 µA µm ^−1^cm^−2^. The GO–C_60_ hybrid‐based sensor also showed a wide linear response and high selectivity along with good practicality toward the detection of DA in rat brain and commercial DA injection samples.^88^

In addition to the applications of fullerene–graphene hybrids in solar cells and electrochemical sensors, fullerene–graphene hybrids have also been applied in supercapacitors. In 2014, Qin and co‐workers constructed a C_60_/graphene hybrid by using a simple solution reaction of GO and C_60_ in a lithium hydroxide (LiOH) saturated solution.^31^ C_60_ particles on the GO surface served as spacers in the C_60_/graphene hybrid to support the graphene sheets. The noncovalent C_60_/graphene hybrid was employed as electrodes in a supercapacitor affording a capacitance of 135.36 F g^−1^ at a current density of 1 A g^−1^, which was higher than that based on pure graphene electrodes (101.88 F g^−1^). Furthermore, a remarkable retention rate of 92.35% after 1000 charge/discharge cycles was obtained, indicating the potential of the C_60_/graphene hybrid as a supercapacitor electrode.^31^

Apart from physical blending, a new approach for constructing fullerene–graphene noncovalent hybrids was developed by Gournis and co‐workers in 2015. The approach was based on a bottom‐up layer‐by‐layer process, which proceeded via the formation of a hybridized organo‐GO Langmuir film, followed by the intercalation of C_60_ molecules by self‐assembly and the Langmuir–Schaefer deposition technique.^89^ The structure of the C_60_–GO hybrid deposited on hydrophobic substrates was characterized by X‐ray diffraction, Raman and X‐ray photoelectron spectroscopies, atomic force and scanning electron microscopies, and conductivity measurements, confirming that C_60_ indeed existed in the hybrid multilayer system and that intercalation of fullerene into the interlayer space of the organo‐GO nanosheets did not affect the structure of GO itself. More importantly, a considerable improvement of an order‐of‐magnitude increase in the electrical conductivity for the C_60_–GO hybrid was achieved because of the presence of C_60_ intercalated between the graphene layers.^89^

#### 2.1.3 Noncovalent Hybrids of C_60_ Derivatives and Graphene

Different from the pristine fullerenes, fullerene derivatives bear versatile functional groups grafted via chemical functionalization, facilitating their assembly on different substrates, including graphene films. In 2012, Kim, Lee and co‐workers constructed a C_60_–graphene hybrid in which alkylated C_60_ molecules self‐organized into nanoscale flake‐like films on a CVD‐grown graphene layer.^29^ The C_60_–graphene hybrid devices exhibited a clear and highly reproducible change in conductance upon visible‐light illumination due to the hole generation in the alkylated C_60_ molecules and the transfer of the holes to the graphene, as deduced from the upshift in the Dirac point and the broadening of the resistance. Moreover, a hole‐doping effect was observed in the C_60_–graphene hybrid devices, with carrier mobility decreasing from 286 to 208 cm^2^ V^−1^ s^−1^ upon illumination with visible light.^29^

**Figure 6:**
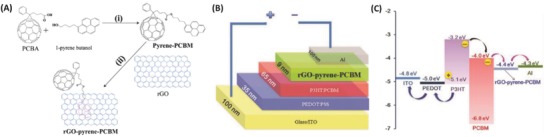
A) Synthetic routes of rGO–pyrene–PCBM. (i) DCC and DMAP, CS_2_:CH_2_Cl_2_ (1:1 v/v); RT; (ii) hydrazine and ammonia, DMF; 95 °C; the B) schematic architecture and C) energy level diagram of ITO/PEDOT:PSS/P3HT:PCBM/rGO–pyrene–PCBM/Al BHJ‐PSC device with the incorporation of rGO–pyrene–PCBM electron extraction layer. The thickness of each layer was given in the left panel. Reproduced with permission.^28^ Copyright 2013, American Chemical Society.

In 2013, our group successfully attached a C_60_ derivative based on [6,6]‐phenyl‐C_61_‐butyric acid methyl ester (PCBM) to rGO via the noncovalent approach in which pyrene was used as an anchoring bridge to link the rGO and PCBM components (**Figure** **6**A).^28^ The synthesized rGO–pyrene–PCBM hybrid showed greatly improved dispersity in *N*,*N*‐dimethylformamide (DMF) owing to the intramolecular *π–π* stacking interactions between the graphene sheet and pyrene–PCBM. Importantly, according to the proposed geometric configuration of rGO–pyrene–PCBM, the C_60_ moiety was far from the graphene sheet and was bridged to the graphene sheet via the pyrene anchor. The rGO–pyrene–PCBM hybrid was then applied as an electron‐extraction material in P3HT:PCBM bulk heterojunction polymer solar cell (BHJ‐PSC) devices (Figure 6B), leading to an efficiency enhancement of ≈15% relative to that of the reference device without an electron‐extraction layer. This result was interpreted as the matching of the work function of the rGO–pyrene–PCBM hybrid with the LUMO level of the PCBM acceptor (Figure 6C), benefiting the formation of an ohmic contact between the Al cathode and the P3HT:PCBM active layer.^28^

**Figure 7:**
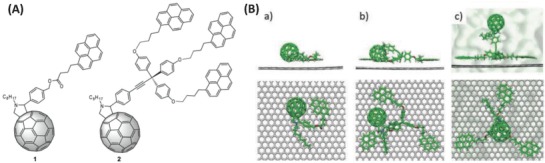
A) C_60_ derivatives endowed with mono (1) and tripodal (2) pyrene units. B) Side and top views of the minimum‐energy geometry calculated at the molecular mechanics level for a representative structure of the supramolecular assembly of graphene with 1 (B‐a) and 2 (B‐b) in the gas phase, and with 2 in the presence of the solvent (B‐c). Reproduced with permission.^90^ Copyright 2017, the Royal Society of Chemistry.

Using a similar strategy, in 2015, Martin and co‐workers synthesized two pyrene functionalized fullerene derivatives via a 1,3‐dipolar cycloaddition reaction between the corresponding pyrene‐based aldehydes with *N*‐octylglycine and C_60_ (**Figure** **7**A).^90^ With pyrene acting as an anchoring bridge, fullerene–graphene hybrids were successfully constructed by sonicating the mixture of pyrene‐functionalized fullerene derivatives suspended in a graphene dispersion exfoliated in *N*‐methylpyrrolidone (NMP). The configurations of the fullerene–graphene hybrids were studied by molecular mechanics/molecular dynamics (MD) calculations, predicting that fullerene derivatives favorably interacted with graphene through both the pyrene base and the C_60_ moiety (Figure 7B).^90^

Guo and co‐workers developed a new strategy to synthesize a C_60_@graphene hybrid, which was uniformly dispersed in a polypropylene (PP) matrix for improved thermal and mechanical properties. By using GO as the starting material, they prepared the C_60_@graphene hybrid (C_60_: ≈47.9 wt% and graphene: ≈35.1 wt%) via a three‐step reaction, with highly active amino groups introduced to react with C_60_ and contribute to the subsequent in situ reactive compatibilization reaction to improve the dispersion of the hybrid. The incorporation of 2.0 wt% C_60_@graphene (relative to PP) remarkably increased the initial degradation temperature by ≈59 °C and simultaneously enhanced the tensile strength and Young's modulus by 67% and 76%. The improvement in the thermal stability of PP was speculated to be due to the free‐radical‐trapping effect of C_60_ and the thermal barrier effect of the graphene layers, while the mechanical enhancement was mainly attributed to the reinforcing effect of the graphene nanosheets and the effective load transfer from the polymer matrix to the graphene sheets.^91^

#### 2.1.4 Noncovalent Hybrids of Endohedral Fullerene and Graphenes

Different from hollow fullerenes, such as C_60_, endohedral fullerenes (EMFs) with metal ions or metallic clusters entrapped in the carbon cage have been attracting considerable attention during the last two decades due to the charge transfer from the internal species to the outer carbon cage.^3^, ^92^ Among the known EMFs, the metal nitride clusterfullerene Sc_3_N@*I_h_*‐C_80_ is so far the most abundant and has an excellent stability superseding other conventional EMFs.^93^ In particular, the unique electronic configuration of (M_3_N)^6+^@(C_2n_)^6−^, resulting from the six‐electron transfer from the endohedral species to the carbon cage, renders an enhanced chemical reactivity and adsorption energy on surfaces compared with that of hollow fullerenes.^94^ An experimental report on a hybrid of an endohedral fullerene and graphene has yet to be published. In 2016, Zope et al. reported a DFT theoretical study on the geometric and electronic structures of Sc_3_N@C_80_ absorbed on pristine graphene nanoflakes (GNFs). The Sc_3_N@C_80_–graphene noncovalent hybrid model was constructed, for which the simulated graphene nanoflake, composed of 96 C and 24 H atoms (C_96_H_24_) with symmetric hexagonal units, served as a support for the deposition of fullerenes in the center.^94^ Compared to C_60_, which adsorbed on GNFs with an energy of 0.76 eV, Sc_3_N@C_80_ adsorption on the GNF resulted in a higher binding energy of 1.00 eV for nearly degenerate isomers that have a pentagon and a hexagon facing the single‐layer graphene (SLG). Such a large binding energy was explained in terms of a higher dispersion interaction between Sc_3_N@C_80_ and the GNF, and the fact that charge separation in Sc_3_N@C_80_ favored binding with the GNF.^94^ Since a large variety of EMFs with different types of endohedral species transferring different numbers of electrons to the carbon cage have been isolated,^3^, ^95^ experimental studies of hybrids of endohedral fullerenes with graphene are eagerly needed and expected to fulfill unique properties that are not accessible by hollow fullerenes.

### 2.2 Covalent Hybrids of C_60_ and Graphenes

Although the noncovalent approach has been extensively used to construct fullerene–graphene hybrids, being facile and effective in achieving improved properties relative to the single components of fullerene and graphene, the determination of their chemical structures is difficult, making the manipulation of their structures and properties unrealistic. Additionally, the noncovalent nature renders relatively weak intermolecular interactions between fullerenes and graphenes. Thus, the covalent hybridization of fullerene and graphene is highly desired to construct hybrids in a controllable manner.^96^ Upon attaching fullerene molecules onto graphenes via covalent bonds, the covalent hybrids afford stronger intermolecular interactions between fullerene and graphene moieties and are more promising than the noncovalent hybrids in terms of the structural modulation of graphene. Covalent hybridizations of fullerenes and graphenes have been accomplished by limited methods, as listed in **Table** **2**.

**Table 2:** Covalent hybrids of fullerenes and graphenes

Type	Graphene raw material	Preparation method	Application/performance	Ref.
Pristine fullerene‐graphene	Pristinea)	Lithiation reaction	Polymer solar cells/PCE: 0.49%	103
	Graphite	Ball‐milling	Oxygen reduction reaction/limiting current: 3.4 mA cm^−2^	104
	Graphite	Thermal treatment	Selective organic derivatization	105
Fullerene derivative‐graphene	GOb)	Coupling reaction	–	32
	GO	Coupling reaction	Nonlinear optical	110
	GO	Esterification	Nonlinear optical	111
	GO	Esterification	–	33
	GO	Esterification	Ultrafast charge separation dynamics	112
	GO	Alkyne‐azide cycloaddition reaction	Solar cells	34
	GO	Esterification	Photoinduced electron transfer	20
	GO	Nucleophilic addition	Microelectromechanical systems	35
	GO	Nucleophilic addition	Photodynamic therapy	114

^a)^ Pristine graphene was synthesized by either chemical vapor deposition (CVD) or exfoliation of graphite

^b)^ Graphene oxide (GO) was prepared by a modified Hummers method.

#### 2.2.1 Covalent Hybrids of Pristine C_60_ and Graphene

**Figure 8:**
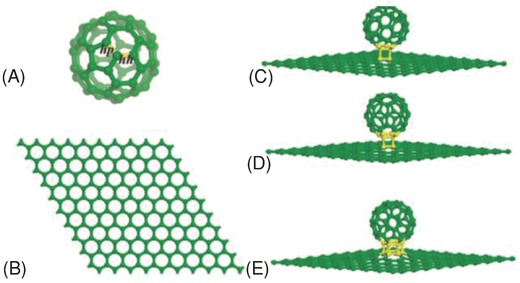
Optimized structures of A) a single C_60_ molecule, B) the supercell of graphene monolayer, the supercell of C) 22‐hp, D) 22‐hh, and E) 66 PGNBs. The binding region between C_60_ and graphene is highlighted in yellow. Reproduced with permission.^17^ Copyright 2009, American Chemical Society.

In 2007, Kauppinen et al. reported the first covalently bonded fullerene‐single‐walled carbon nanotube (SWNT) hybrid, termed as NanoBuds, prepared in continuous aerosol (floating catalyst) reactors by using particles grown in situ via ferrocene vapor decomposition or by using premade iron‐catalyst particles produced by a hot‐wire generator. The authors proposed that fullerenes were formed on the iron‐catalyst particles together with SWNTs during CO disproportionation.^97^ Stimulated by such a seminal work, in 2009, Zeng and co‐worker proposed theoretically an unprecedented covalent hybrid of C_60_–graphene, termed as periodic graphene nanobuds (PGNBs) in which C_60_ molecules covalently attach to a graphene monolayer and form a periodic lattice structure (**Figure** **8**).^17^ Since there are two different types of C—C bonds ([6,6] and [5,6] bonds, labeled as hh and hp, respectively, in Figure 8a) in C_60_, upon covalent attachment onto the graphene monolayer, the binding energy required for different types of hybridizations should be considered. According to the first‐principles DFT results, 22‐hh PGNB was more stable than the other two PGNBs, since it required the least binding energy. Furthermore, various forms of PGNB were all thermally stable at ≈800 K and were either semiconducting or semimetallic, depending on the pattern of chemical bonding between C_60_ and graphene.^17^ Due to the highly curved structure of the nanobuds combined with a decreased work function, the hybrid PGNB is expected to be a promising field‐emission material. In 2013, Ganji et al. studied the lithium adsorption property of PGNBs by means of DFT calculations. The calculated results showed that the binding energy was −2.58 eV when Li bonds to a PGNB at the hollow site above the center of the nonagon ring, which was substantially lower than that for a Li atom on pure graphene (−0.85 eV) and was attributed to a significant charge transfer from the Li to the PGNB. Such strong interactions between the Li atom and PGNB were due to the presence of a nonagon ring (defective site) in the PGNB.^98^ Furthermore, the C_60_ in the hybridized system provided strong adsorption sites and charge transfer, which allowed the other component in the hybridized system to be positively charged as a result of the high electron affinity, leading to the enhanced Li adsorption capabilities of the hybrid PGNB.^98^

Likewise, in 2011, Li et al. designed four kinds of all‐carbon graphene–fullerene nanobuds and studied their magnetic properties by spin‐polarized DFT.^99^ To build up such nanobuds, a C_54_ structure was proposed by removing a six‐membered ring from C_60_. Four different kinds of connection positions for the C_54_ structure on graphene were investigated. In two cases, where six carbon atoms of graphene formed covalent bonds with the fullerene, the graphene–fullerene nanobuds were ferromagnetic at their ground states with magnetic moments of nearly 6 μ_B_; whereas, for the other two cases, nonmagnetic states were found since the connections of two atoms in different sublattices of graphene could not generate unpaired spins.^99^ These predications suggest the possibility of manipulating the physical properties of a graphene–fullerene hybrid via simple control of its hybridization style.

Deymier et al. adopted the self‐consistent charge density functional tight‐binding (DFTB) and DFT methods to evaluate energy barriers for the formation of a single‐layer graphene−C_60_ hybrid.^100^ The calculated results indicated that the C−C distance was 3.0 Å for the relatively weak π–π stacking between the adjacent benzene rings of the fullerene and graphene. When fullerene was bonded to single‐layer graphene with a [2 + 2]‐addition pattern, the newly formed two σ C−C bonds had a length of 1.62 (DFTB) or 1.61 Å (DFT). Meanwhile, the hybrid graphene sheet exhibited a noticeable local curvature at the site of the new C−C bond, which was identical for both DFTB‐optimized and DFT‐optimized structures. Furthermore, the calculated results showed that chemical attachment of the fullerene onto carbon structures with high intrinsic bond angles may have relatively low activation energy barriers. During the process of fullerene bonding, the local curvature of the graphene sheet adjusted by local bond rotation/puckering to optimize the π‐orbital overlap and C−C bond angle, facilitating the formation of the covalent hybrid. Additionally, introducing defects into graphene was another effective strategy to distort its structure, which facilitated a reduction in the activation energy barrier toward fusion with fullerene. As to the site‐selective behavior, attaching C_60_ onto the carbons at the edges of the defect did not have priority over attaching to a pristine graphene sheet because the distortion of the ideal π system in graphene perturbed the *π–π* stacking interactions with the fullerene and forced the fullerene to avoid the defective surface of graphene.^100^

In 2015, Kirca designed a novel fullerene–graphene hybrid composed of fullerene covalently sandwiched between parallel graphene sheets by using MD simulations.^101^ They established the atomistic models for the fullerene–graphene hybrid by employing different fullerene types (i.e., C_180_, C_320_, C_540_, and C_720_) as sandwich cores. According to their calculation results, dangling bonds on the reciprocal locations of both graphene and fullerenes promoted the formation of covalent bonds between the fullerene and graphene layers. Moreover, the thermodynamic stability of the fullerene–graphene hybrid was evaluated with potential energy profiles, and the total configuration energies for all systems were minimized and remained stable over a long period of time, suggesting good thermodynamic stability.^101^ The author's simulation results also showed that the size of the fullerene sandwiched between the graphene layers had a notable influence on the compressive behavior of the hybrid and on the energy absorption capacity.^101^ In 2016, the same group further investigated theoretically the hydrogen storage capacity of such a sandwiched fullerene–graphene hybrid and proposed that the hydrogen storage performance of the fullerene–graphene hybrid could be improved by the appropriate selection of fullerene types. Grand canonical Monte Carlo calculations at a temperature of 77 K and up to 1 bar pressure conditions demonstrated that the hydrogen storage capacity of the hybrid surpassed 5 wt%. Furthermore, the calculated results showed that lithium doping for the sandwiched fullerene–graphene hybrid effectively improved the hydrogen adsorption capacity under the same conditions.^102^

**Figure 9:**
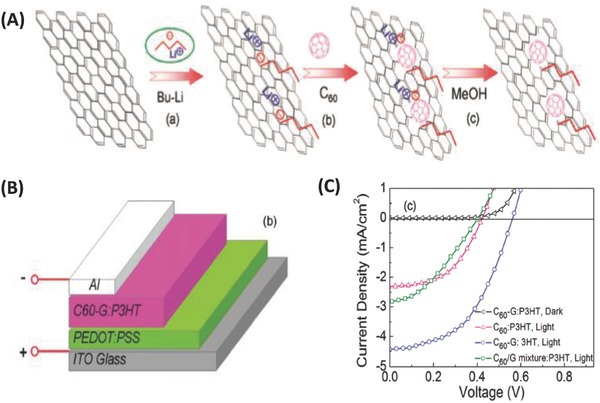
A) Schematic representation of grafting C_60_ onto graphene through lithiation reaction with n‐butyllithium. B) Schematic of a hybrid photovoltaic device with the C_60_‐G:P3HT composite as the active layer, C) J–V curves of the photovoltaic devices with the C_60_‐G:P3HT (1:1 wt/wt), the C_60_:P3HT (1:1 wt/wt), or the C_60_/G mixture (12 wt% G):P3HT (1:1 wt/wt) as the active layers after annealing treatment (130 °C, 10 min). Reproduced with permission.^103^ Copyright 2011, American Chemical Society.

The experimental synthesis of a fullerene–graphene covalent hybrid was not reported until 2011, when Dai et al. developed a simple lithiation method to covalently attach monosubstituted C_60_ onto graphene nanosheets (**Figure** **9**A). In the first step, graphene was treated with n‐butyllithium, creating a “living” center of lithium where the nucleophilic addition of C_60_ occurred. According to the TEM results, the monosubstituted C_60_ moieties acted as nucleation centers to promote the formation of C_60_ aggregates ≈5 nm in diameter on the graphene surface. The resultant C_60_–graphene covalent hybrid was used as an electron acceptor in P3HT‐based bulk heterojunction solar cell devices (Figure 9B), showing significantly improved electron transport, and consequently a PCE of 1.22% was obtained (Figure 9C), which was 2.5‐fold higher than that of the C_60_:P3HT counterpart.^103^ The enhanced short‐circuit current density (*J*_sc_) was attributed to the improved electron transport because the introduction of the C_60_–graphene covalent hybrid could provide a direct conduction path for electron transport via percolation through the highly conducting 2D graphene sheets.^103^

**Figure 10:**
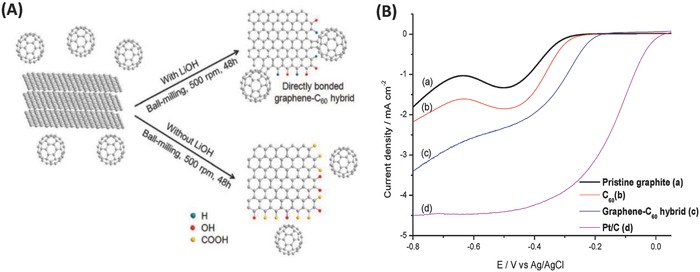
A) Schematic illustration of the mechanochemical reaction between graphite and C_60_ in a sealed ball‐mill crusher. The synthesis routes with LiOH affording the directly bonded graphene–C_60_ hybrid or without LiOH leading to the formation of oxygenated graphene nanoplatelets and unreacted C_60_ are both shown. B) Linear sweep voltammograms (LSVs) of the pristine graphite (a), C_60_ (b), the graphene–C_60_ hybrid (c), and commercial Pt/C (d) in an O_2_‐saturated 0.1 m aqueous KOH solution at a scan rate of 10 mV s−^1^ with a rotation rate of 1600 rpm. The current density is based on the geometric area (0.2 cm^2^) of the GC disk electrode. Reproduced with permission.^104^ Copyright 2015, the Royal Society of Chemistry.

A breakthrough in the experimental synthesis of fullerene–graphene covalent hybrids was made by our group in 2015.^104^ Stimulated by the effectiveness of solid‐state mechanochemical edge‐selective functionalization of graphene via ball milling, we successfully synthesized the first directly bonded C_60_–graphene hybrid by ball milling in which C_60_ was directly bonded to the edges of graphene nanoplatelets via two C—C single bonds, as confirmed by high‐resolution transmission electron microscopy (HR‐TEM) (**Figure** **10**A).^104^ Furthermore, the directly bonded C_60_–graphene hybrid was applied as a carbon‐based electrocatalyst toward the oxygen reduction reaction (ORR), showing improved ORR electrocatalytic activity over the pristine graphite in terms of a higher limiting current (−3.4 mA cm^−2^) than those of the pristine graphite (−1.8 mA cm^−2^) and C_60_ (−2.2 mA cm^−2^). This was interpreted as the charge transfer from graphene to C_60_ enabling the graphene basal plane to be positively charged and thus to be more electrocatalytically active toward the ORR than the pristine graphite (Figure 14, I–IV).^104^

**Figure 11:**
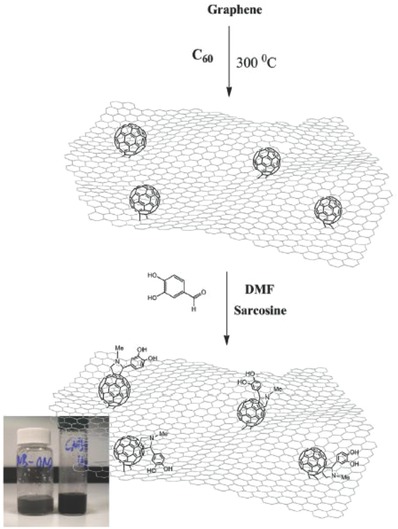
Schematic representation of the 1,3 dipolar cycloaddition selectively on the C_60_ rings of the pG‐C_60_‐f‐OH product. The inset photo shows a diluted and a concentrated dispersion of pG‐C_60_‐f‐OH in water (2 mg mL^−1^). The concentrated dispersion is stable without any signs of precipitation for several days. Reproduced with permission.^105^ Copyright 2016, Elsevier B.V.

In 2016, Georgakilas et al. reported the preparation of “pure” graphene nanobuds via a simple thermal treatment of a homogenous mixture of pG nanosheets and pristine C_60_. After heating the pG/C_60_ mixture composed of a saturated solution of C_60_ in 1‐chloronaphthalene mixed with pG nanosheets at 300 °C for 2 h, C_60_ was impregnated into pG nanosheets with the evaporation of the solvent, affording the formation of the pG–C_60_ covalent hybrid (**Figure** **11**).^105^ The pG–C_60_ covalent hybrid showed remarkable dispersability, a decreased number of defects, and a highly aromatic character contributing to an improved electrical conductivity compared to that of pristine graphene. Furthermore, the authors carried out a selective organic functionalization of the pG–C_60_ covalent hybrid via a 1,3‐dipolar cycloaddition using azomethine ylide and determined the as‐synthesized organically modified pG–C_60_–f–OH hybrid preserved the electronic properties of the pG–C_60_ hybrid and showed a similar electrical conductivity. These results indicate that the all‐carbon hybrid strategy of pG–C_60_ provides a simple way to increase the reactive sites of the graphene surface without damaging the aromatic character of graphene.^105^

#### 2.2.2 Covalent Hybrids of Fullerene Derivatives and Graphene

The high chemical reactivity of fullerene makes derivatization of fullerenes feasible, and to date, a large number of fullerene derivatives have been synthesized via several well‐established routes, such as Prato, Bingel–Hirsch, Diels–Alder, radical reaction, azide addition, nucleophilic addition, cycloaddition, hydroxylation reactions, and carbene addition.^106^, ^107^, ^108^, ^109^ Unlike pristine fullerene, with limited solubility in organic solvents, upon derivatization the grafted functional groups onto the fullerene's cage not only improves the solubility of fullerene but also provides additional reaction sites for further covalent hybridization with graphene. Therefore, covalent hybrids of fullerene derivatives with graphene have been extensively studied during the past decade.

**Figure 12:**
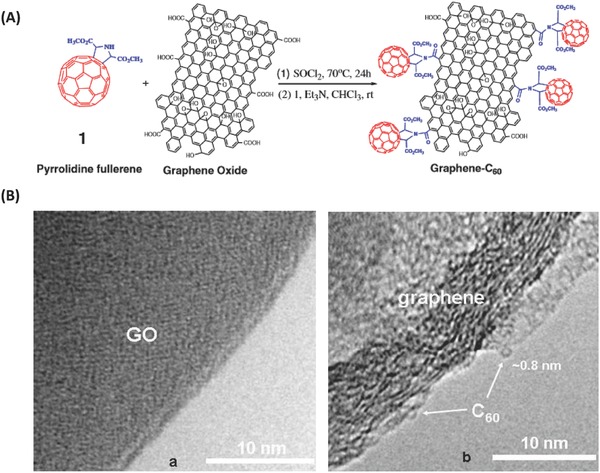
A) Synthesis procedure of the C_60_ derivative‐graphene hybrid. B) HR‐TEM images of GO (B‐a) and graphene–C_60_ hybrid (B‐b). The images were acquired from samples deposited onto a holey carbon support film. Reproduced with permission.^32^ Copyright 2008, Elsevier B.V.

The first covalent hybrid of a C_60_ derivative with graphene was reported by Chen and co‐workers in 2008 via a chemical coupling reaction between GO and pyrrolidine fullerene (**Figure** **12**A).^32^ Pyrrolidine fullerene was prepared by a photochemical reaction between amine acid esters and C_60_, and GO was prepared via a commonly used and modified Hummers' method, followed by acyl‐chloride functionalization. Finally, a condensation reaction between pyrrolidine fullerene and the acyl‐chloride functionalized GO was carried out, affording the C_60_ derivative‐graphene hybrid. The covalent linkage between pyrrolidine fullerene and graphene oxide was verified by Fourier‐transform infrared spectroscopy (FTIR), thermal gravimetric analysis (TGA), and HR‐TEM (Figure 12B). According to the X‐ray photoelectron spectroscopy (XPS) characterization results, on average, one C_60_ molecule was covalently attached for every ≈130 carbon atoms of the C_60_ derivative‐graphene hybrid.^32^ In a follow‐up study, the authors studied the nonlinear optical property of the C_60_ derivative‐graphene hybrid by using the Z‐scan technique at 532 nm on nanosecond and picosecond timescales. They found that covalently functionalizing graphene with the fullerene, as the reverse saturable absorption chromosphere, enhanced the nonlinear optical performance in the nanosecond regime. This result was because of the combination of effects of a nonlinear mechanism and the photoinduced electron or energy transfer between the fullerene moiety and graphene.^110^

Alternatively, using a facile esterification reaction, the same group prepared a novel C_60_ derivative‐graphene hybrid based on the esterification of fullerenol and GO.^111^ The estimated content of C_60_ molecules covalently attached was ≈110 carbon atoms in graphene per one C_60_ molecule, according to UV and elemental analysis measurements. Furthermore, the enhanced nonlinear optical property of the C_60_ derivative‐graphene hybrid relative to its single components of C_60_ and graphene, measured by open‐aperture Z‐scan, was attributed to the photoinduced electron transfer between the graphene sheet and C_60_.

**Figure 13:**
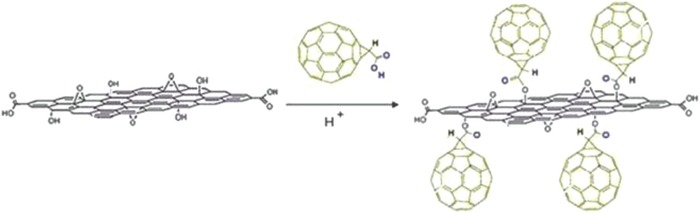
Functionalization of the graphite oxide via the Fisher esterification. Reproduced with permission.^33^ Copyright 2011, the Royal Society of Chemistry.

While C_60_ derivatives mainly attached at the edges of GO within the above C_60_ derivative‐graphene hybrids, it is intriguing to address whether C_60_ derivatives can be grafted to the surface of a graphene sheet. In 2011, Wang and co‐workers developed a new strategy to graft a fullerene derivative onto the graphene surface via an esterification reaction between fullerenoacetic acid and GO.^33^ As a result, fullerene molecules were inserted in between graphene layers and grafted on both sides of graphene (**Figure** **13**), and fullerene behaved as a space impediment to prevent the re‐stacking of exfoliated graphene, thus benefiting the exfoliation of graphene sheets.^33^

**Figure 14:**
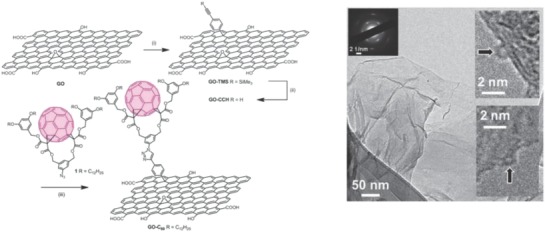
Left: Preparation of GO–C_60_. Right: HR‐TEM images of GO–C_60_ and its corresponding SAED pattern. Insets on the right show spherical C_60_ units highlighted with arrows. Reproduced with permission.^34^ Copyright 2014, the Royal Society of Chemistry.

In 2014, Langa and co‐workers synthesized a photoresponsive covalent hybrid of a fullerene derivative and functionalized GO by using a click reaction. GO was first modified with 4‐(trimethylsilyl)‐ethynylaniline via an aryl diazonium salt reaction, followed by cleavage of the trimethylsilyl groups using tetra‐*n*‐butylammoniumfluoride to afford GO‐CCH. Then, a Cu‐catalyzed alkyne‐azide cycloaddition reaction was carried out to graft the fullerene building block (**1**) onto the GO‐CCH scaffold (**Figure** **14**).^34^ Raman spectroscopic results confirmed that the click cycloaddition reaction between the fullerene derivative and GO‐CCH occurred via the acetylenic bond of GO‐CCH rather than directly onto the GO surface. The successful synthesis of the fullerene derivate‐GO‐CCH hybrid was confirmed by HR‐TEM results, showing the existence of the spherical structure of fullerene moieties, with an inner diameter of ≈0.7 nm, grafted onto GO (Figure 14). Moreover, transient absorption spectra showed that long‐lived transient species of C_60_^•−^ originated from the photoinduced intramolecular electron transfer from GO to C_60_.^34^

**Figure 15:**
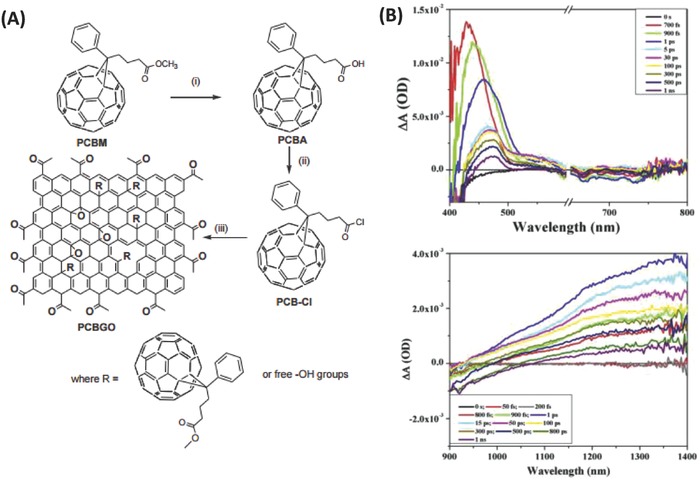
A) Synthesis of PCBA and PCBGO. B) Ultrafast transient absorption spectra of PCBGO in THF in (top) visible (break is shown from 600 to 650 nm for pump double resonance at 640 nm) and (bottom) NIR region on excitation with 320 nm pump wavelength. Reproduced with permission.^112^ Copyright 2016, Elsevier B.V.

Later, by using hydrolyzed PCBM as a fullerene precursor via a very mild process without the use of metal catalysts, Kumar et al. synthesized a novel fullerene–GO covalent hybrid (PCBGO) by grafting PCBM onto GO via flexible linkages (**Figure** **15**).^112^ The obtained PCBGO is a typical donor–acceptor molecule, where the graphene oxide sheets, with long range ordering and high electron density, acted as the donor moiety, while the fullerenes acted as the electron–acceptor moiety. Ultrafast transient absorption spectra showed a very slow charge recombination rate of 1.5 × 10^9^ s^−1^ for C_60_^•−^, suggesting the efficient charge separation and formation of long‐lived charge separated species.^112^

**Figure 16:**
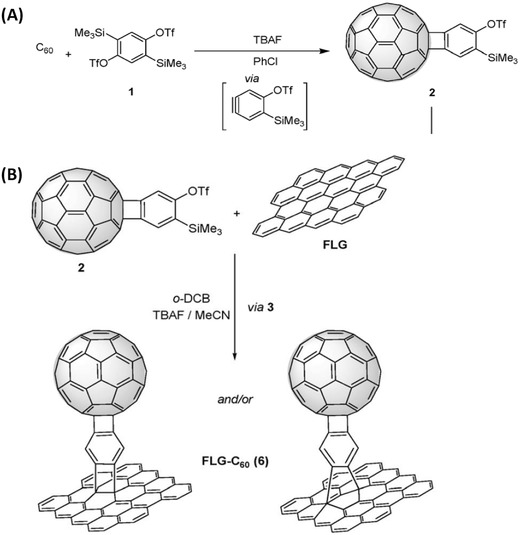
A) Synthesis of monoadduct **2** via [2 + 2] cycloaddition. B) Cycloaddition reaction of few layered graphene (FLG) and corresponding structures. Reproduced with permission.^18^ Copyright 2016, the Royal Society of Chemistry.

In 2016, Martin and co‐workers, via a two‐step reaction, synthesized a novel fullerene–graphene hybrid by using phenyl as a spacer.^18^ C_60_ reacted with 2,5‐bis(trimethylsilyl)‐1,4‐phenylene bis(triflate) via a [2 + 2] cycloaddition yielding a C_60_‐aryne precursor (**Figure** **16**A), which reacted further with pristine graphene. The obtained fullerene–graphene hybrid was characterized by TGA, FTIR, and Raman spectroscopies, XPS, atomic force microscopy (AFM), and TEM. According to theoretical calculations, the hybrid was proposed to be a [4 + 2] cycloadduct, which was the more stable configuration to be formed (Figure 16B), while the formation of [2 + 2] cycloadducts also seemed possible because of the small difference in energy.^18^

**Figure 17:**
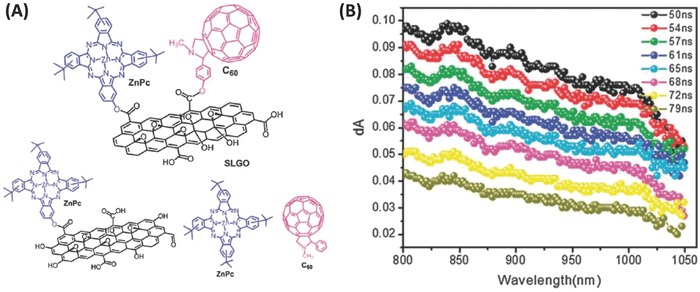
A) Structures of the ZnPc–SLGO–C_60_ hybrid and the control compounds. B) Nanosecond transient absorption spectra of the ZnPc–SLGO–C_60_ hybrid at different time intervals after excitation with 532 nm laser of 7 ns pulse width in DMF. Reproduced with permission.^19^ Copyright 2013, the Royal Society of Chemistry.

In addition to above‐mentioned binary fullerene–graphene hybrids, the inclusion of another functional material to form ternary hybrids with fullerene and graphene as building blocks has also been reported. In 2013, Souza and co‐workers reported a novel ternary covalent fullerene–graphene hybrid ZnPc–SLGO–C_60_ in which zinc(II) phthalocyanine (ZnPc), acting as electron donor, and fulleropyrrolidine, acting as electron acceptor, were covalently linked onto single‐layer graphene oxide (SLGO) simultaneously (**Figure** **17**).^19^ The covalent hybrid material was characterized by UV–vis, fluorescence, TEM, Raman, TGA, and electrochemistry. Furthermore, transient spectroscopic results showed that a photoinduced electron transfer occurred from ZnPc to ^1^C_60_*, with the lifetime of the charge‐separated state being ≈0.04 µs, to afford the radical ion pair (ZnPc^•+^‐SLGO–C_60_^•−^), as evidenced by the appearance of the cation (ZnPc^•+^) and anion (C_60_^•−^) radicals in the visible to near‐infrared (NIR) regions. In contrast, for the control samples, GO and binary hybrids ZnPc–GO and GO–C_60_, no cation and anion radicals were observed. Thus, the formation of the ternary hybrid is crucial for such a photoinduced electron transfer phenomenon.^19^

In 2014, Wang and co‐workers fabricated a graphene–C_60_ hybrid lubricating film by a multistep self‐assembly method on silicon surfaces.^35^ They first prepared a self‐assembled monolayer of (3‐aminopropyl)‐triethoxysilane (APTES) covalently anchored onto the silicon wafer via Si—O—Si covalent bonding, followed by covalently attaching GO onto the APTES‐self‐assembled monolayer (SAM) surface through chemical reactions between epoxy‐carboxyl and amine groups. To graft C_60_, the GO films assembled on the silicon wafers were amine‐functionalized by (*N*‐[3‐(trimethoxysilyl)‐propyl]‐ethylenediamine) molecules, which contained primary and secondary amino groups capable of undergoing N—H addition reactions across the C=C bonds in C_60_. The successful grafting of C_60_ chemically to the amine‐functional GO by nucleophilic addition reactions was verified by FTIR, X‐ray powder diffraction (XRD), TGA, Raman spectroscopy and XPS. Colloidal probe measurements show that APTES‐a‐graphene–C_60_ exhibited a low nanofriction coefficient due to the existence of a more ordered C_60_ outer layer, which facilitated microsphere sliding because of the small contact area between the microspheres and films, in addition to the presence of the few‐layer graphene with excellent nanolubrication properties. Furthermore, the conclusion pointed out that the strong bonds, with stable and not easily damaged characteristics, between the carbon atoms of C_60_ facilitated the long‐lasting and effective lubrication.^35^

**Figure 18:**
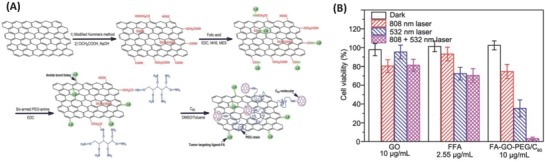
A) The preparation of the FA–GO–PEG/C_60_ nanohybrid. B) Intracellular ROS generation by GO, FFA, and FA–GO–PEG/C_60_. HeLa cells are exposed to 808 nm laser (light dose: 360 J cm^−2^), 532 nm laser (light dose: 30 J cm^−2^), or 808 and 532 nm laser for light irradiation, respectively. Reproduced with permission.^114^ Copyright 2015, the Royal Society of Chemistry.

Graphenes have been reported to act as an excellent drug carrier and photothermal agent, which can efficiently convert NIR light into heat and thus induce hyperthermia to cells and surrounding tissues.^113^ To extend the biomedical applications of graphenes, water‐soluble functional groups may be incorporated into fullerene–graphene hybrids, and the covalent hybrids between fullerene and graphene can effectively prevent the spontaneous aggregation of the C_60_ or graphene, rendering them stable in solution with the free movement of C_60_ coated on the graphene restricted.^113^ In 2015, Hu and co‐workers synthesized a multifunctional graphene–C_60_ covalent hybrid incorporating folic acid (FA) and polyethylene glycol (PEG) onto GO via an imide linkage (**Figure** **18**A). The formation of the as‐synthesized FA–GO–PEG/C_60_ hybrid dramatically improved tumor targeting, as demonstrated by a cellular uptake assay.^114^ According to the photodynamic and photothermal measurements of the FA–GO–PEG/C_60_ hybrid, the photothermal conversion efficiency showed a considerable photothermal therapy (PTT) effect of 44.4% compared with that for the Au nanoparticles of 30%. Furthermore, when the FA–GO–PEG/C_60_ is taken by tumor cells, it caused heating and significant intracellular reactive oxygen species (ROS) production under light irradiation, leading to a marked decrease in cell survival and to the elevation of oxidative stress (Figure 18B).^114^ Thus, the evident cell damage due to the PTT of FA–GO–PEG/C_60_ may further enhance the photodynamic therapy (PDT) efficacy against cancer cells. FA–GO–PEG/C_60_ showed a combined PDT and PTT treatment with remarkably improved and synergistic effects compared to that of PTT or PDT alone, having potential for a synergistic phototherapy of cancer.

## 3 Hybrids of C_60_ and Graphite‐Like Carbon Nitride (g‐C_3_N_4_)

**Figure 19:**
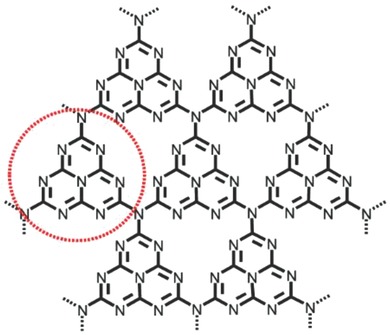
Perfect graphitic carbon nitride (g‐C_3_N_4_) sheet. The red dotted circle marks the basic unit of g‐C_3_N_4_. Reproduced with permission.^116^ Copyright 2012, American Chemical Society.

g‐C_3_N_4_ is the most stable allotrope of carbon nitrides under ambient conditions with the 2D, π‐conjugated plane of polymeric tri‐s‐triazine connected via tertiary amines (**Figure** **19**).^115^ As an emerging graphene‐analogous 2D nanomaterial, g‐C_3_N_4_ also has a high thermal and chemical stability as well as unique electronic and optical properties, making it a promising material for photocatalysis and energy‐related applications.^116^, ^117^ The advantage of g‐C_3_N_4_ as a metal‐free catalyst toward organic pollutants and water splitting for hydrogen production under visible‐light irradiation has been extensively reported.^116^, ^118^ However, the photocatalytic activity of bare C_3_N_4_ is limited due to the high recombination rate of photogenerated electron–hole pairs resulting from its relatively large band gap (≈2.7 eV) and the existence of contact resistance between the nanosheets.^116^ Hence, the photocatalytic activity of g‐C_3_N_4_ needs to be improved so as to extend its photocatalytic applications.

Considering the excellent electron‐accepting ability of C_60_, which is beneficial for a rapid photoinduced charge transfer with donor molecules and a relatively slow charge recombination, hybridizing g‐C_3_N_4_ with C_60_ has been implemented, and the effectiveness of such a strategy in improving the photocatalytic activity of g‐C_3_N_4_ has been examined. In 2014, Chai et al. prepared a g‐C_3_N_4_/C_60_ composite by simply blending g‐C_3_N_4_ powder into a C_60_ solution in toluene.^119^ The resultant g‐C_3_N_4_/C_60_ composites were characterized by XRD, scanning electron microscope (SEM), TEM, and FTIR. The photocatalytic activity of the g‐C_3_N_4_/C_60_ composite in the degradation of rhodamine B was further evaluated, indicating that the g‐C_3_N_4_/C_60_ composite with the optimal amount of C_60_ of 1 wt% had the highest photocatalytic performance. The improved photocatalytic performance of the g‐C_3_N_4_/C_60_ composite was attributed to the synergetic effect of g‐C_3_N_4_ and C_60_, which facilitated the separation of photogenerated electrons and holes.^119^

**Figure 20:**
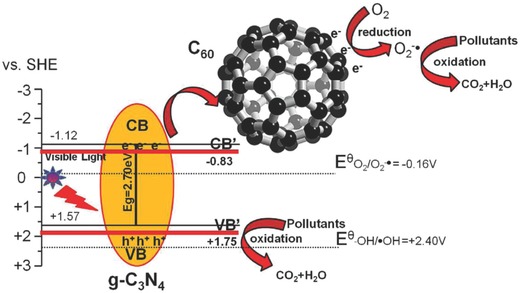
Schematic drawing illustrating synthetic route and the mechanism of charge separation and photocatalytic process over g‐C_3_N_4_ and C_60_/g‐C_3_N_4_ photocatalysts under visible light irradiation. Reproduced with permission.^120^ Copyright 2014, Elsevier B.V.

Another C_60_/g‐C_3_N_4_ composite was prepared by Zhu et al. via the thermal treatment of dicyandiamide, a precursor for synthesis of g‐C_3_N_4_, at 550 °C in atmosphere in the presence of C_60_.^120^ Upon noncovalently hybridizing C_60_ into a matrix of g‐C_3_N_4_, the valance band of g‐C_3_N_4_ shifted to a lower energy position, affording a strong photooxidation capability under visible light. Under the optimal loading amount of C_60_ of 0.03%, the photocatalytic activity of the C_60_/g‐C_3_N_4_ composite for the degradation of phenol and methylene blue under visible light was found to be ≈2.9 and 3.2 times higher than that of pristine g‐C_3_N_4_. The enhanced photocatalytic activity of the C_60_/g‐C_3_N_4_ composite was attributed to the promoted generation of holes, and •OH originated from the strong interaction of the conjugated π‐bond between C_60_ and g‐C_3_N_4_, leading to a rapid photogenerated electron transfer rate and high charge separation efficiency of C_60_ in the C_60_/g‐C_3_N_4_ composite (**Figure** **20**).^120^

**Figure 21:**
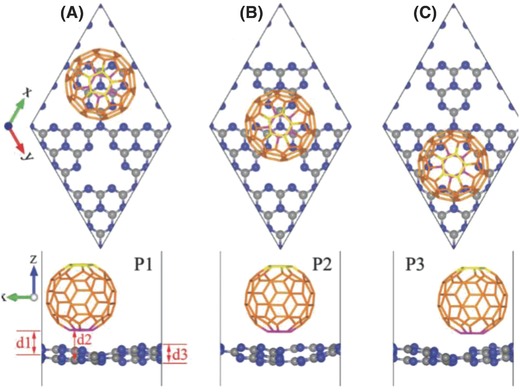
Top (upper panel) and side (lower panel) views of the optimized structures of A) P1, B) P2, and C) P3. Reproduced with permission.^121^ Copyright 2016, the Royal Society of Chemistry.

A fundamental understanding of the corresponding mechanism behind the greatly enhanced photocatalytic activity of g‐C_3_N_4_ by C_60_ modification is needed. In 2016, Xu, Huang and coworkers adopted first‐principles calculations to investigate the interfacial effects of C_60_/g‐C_3_N_4_ nanocomposites on the electronic properties, charge transfer, and optical response in detail.^121^ Three representative central symmetry systems were constructed with the C_60_ molecule center directly over the three labeled points of N1, N3, and M named P1, P2, and P3, respectively, as illustrated in **Figure** **21**. The calculated results showed that (1) the electron transfer mainly happened around the interfaces, (2) the charge transfer in P3 was stronger than those of P2 and P1, and (3) P1 possessed the weakest interfacial interaction among the three systems. A further study unveiled that P3 exhibited the largest charge transfer amount, among which the g‐C_3_N_4_ monolayer loses 0.038 electrons, the C_60_ molecule gains 0.037 electrons, and the rest (0.001 electrons) remained at the interface between them. Interestingly, for different stacking patterns, g‐C_3_N_4_ and C_60_ were always linked by vdW forces and formed type‐II heterojunctions in most cases. The valence band maximum and conduction band minimum of these heterostructures were dominated by the unsaturated nitrogen (N2) atoms and C_60_ molecules, respectively, which strongly interacted with each other and resulted in a strong charge transfer between g‐C_3_N_4_ and C_60_ and a clear bending of the g‐C_3_N_4_ sheets. The unsaturated N2 atoms included at the interfaces have a significant influence on promoting the photocatalytic performance, while the existence of saturated nitrogen (N1 and N3) atoms lying at the interfaces would weaken the interfacial interactions between C_60_ molecules and the g‐C_3_N_4_ monolayers.^121^

Dai et al. reported a systematic theoretical study on the changes in the structure, stability, electronic properties, and light absorption of g‐C_3_N_4_ before and after C_60_ adsorption by using a larger model with 25 different adsorption configurations.^122^ The authors concluded that C_60_ preferred to stay upon the “junction nitrogen” with the carbon atom of fullerene being nearest to the monolayer. The adsorption of fullerene resulted in a change in the geometrical morphology of g‐C_3_N_4_ from flat to wrinkled via an irreversible process. The wrinkled structure was more stable by ≈5.6 eV compared with that of the flat one. The shortest distance between g‐C_3_N_4_ and C_60_ for all configurations was ≈3 Å, indicating the existence of vdW interactions. Furthermore, the analysis of the electronic properties indicated that the adsorption of C_60_ onto g‐C_3_N_4_ did not result in efficient carrier separation at interface. In fact, C_60_ in these systems mainly induced the geometrical distortion of g‐C_3_N_4_ monolayers, thus leading to the improved photocatalytic performance.^122^

In 2017, Cai et al. synthesized C_60_/g‐C_3_N_4_ and C_70_/g‐C_3_N_4_ hybrids via a hydrothermal process.^123^ XRD, UV–vis diffuse reflectance spectroscopy, FTIR, SEM, and HR‐TEM measurements verified the successful hybridization of fullerenes with g‐C_3_N_4_. The photocatalytic disinfection activity of these hybrids on the inactivation of *Escherichia coli* (*E. coli*) O157:H7 showed that the bacterial cell could be damaged by the active oxygen radicals (•O_2_^−^ and •OH) produced from the fullerene/g‐C_3_N_4_ hybrids under visible‐light irradiation.^3^ Additionally, the C_70_/g‐C_3_N_4_ composite exhibited a higher photocatalytic inactivation activity for completely inactivating all *E. coli* O157:H7 cells within 4 h under visible‐light irradiation. The high catalytic performance was attributed to the effective transfer of the photoinduced electrons from the fullerene/g‐C_3_N_4_ hybrids under visible‐light irradiation.

**Figure 22:**
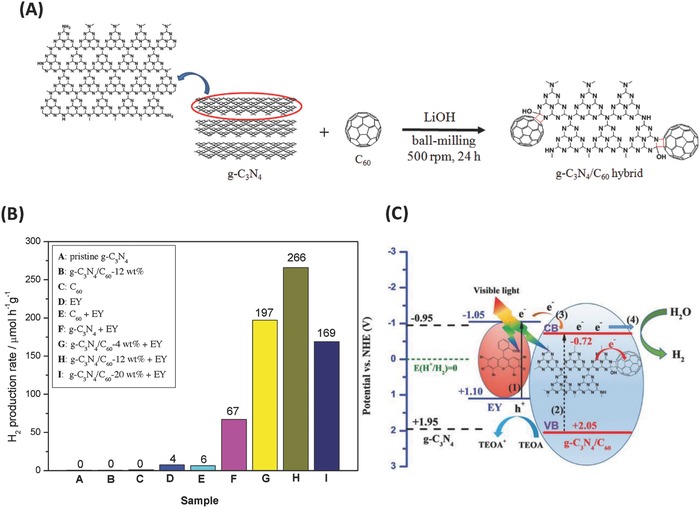
A) The schematic illustration of g‐C_3_N_4_‐C_60_ structure. B) Photocatalytic H_2_ production rates of different samples measured in 5 vol% TEOA aqueous solution in the presence of EY for 3 h under visible light (λ > 420 nm) irradiation of a 300 W Xe lamp. C) A schematic of the photocatalytic H_2_ production mechanism for the g‐C_3_N_4_/C_60_ hybrid. Reproduced with permission.^55^ Copyright 2017, the Royal Society of Chemistry.

All of the aforementioned experimental reports on fullerene/g‐C_3_N_4_ hybrids are based on noncovalent hybridization, for which C_60_ was blended physically into the pristine g‐C_3_N_4_ or its precursor in a low blending ratio (<2.0 wt%), and a large number of unreacted C_60_ are retained in the composites. On the other hand, the photocatalytic activity of the C_60_/g‐C_3_N_4_ composite was examined only via the degradation of dyes. These limitations were not broken until the recent success of our group in synthesizing the first fullerene–g‐C_3_N_4_ covalent hybrid via a facile solid‐state mechanochemical route by ball‐milling g‐C_3_N_4_ and C_60_ in the presence of LiOH as a catalyst (**Figure** **22**A).^55^ The formation of covalent bonds between g‐C_3_N_4_ and C_60_ via a four‐membered ring of azetidine was confirmed by FTIR spectra, Raman spectra, XPS survey spectra, XRD patterns, SEM, TEM, and HR‐TEM. More importantly, we further carried out the first application of a fullerene‐C_3_N_4_ hybrid in visible‐light photocatalytic hydrogen production. As a systematic investigation, a series of g‐C_3_N_4_/C_60_ hybrids were prepared by ball‐milling mixtures of bulk g‐C_3_N_4_ and C_60_ powders in different weight ratios of C_60_:g‐C_3_N_4_ (20:500 mg = 4 wt%, 60:500 mg = 12 wt%, 100 mg:500 mg = 20 wt%, denoted as g‐C_3_N_4_/C_60_‐4wt%, g‐C_3_N_4_/C_60_‐12 wt%, and g‐C_3_N_4_/C_60_‐20 wt%, respectively). Under the optimized conditions, the g‐C_3_N_4_/C_60_‐12 wt% hybrid exhibited the highest H_2_ production rate of 266 µmol h^−1^ g^−1^, which was ≈4.0 times higher than that for the pristine g‐C_3_N_4_ photocatalyst (67 µmol h^−1^ g^−1^, in the presence of eosin Y (EY)) (Figure 22B). The mechanism for the enhanced H_2_ production rate for the g‐C_3_N_4_/C_60_‐12 wt% hybrid was proposed as the conjunct effects of the facilitated electron transfer from the photosensitizer (EY) to g‐C_3_N_4_ due to the lowering of the conduction band of g‐C_3_N_4_ and the inhibited recombination of photoinduced electron–hole pairs owing to the fast photoinduced electron transfer process from g‐C_3_N_4_ to C_60_ (Figure 22C).^55^

## 4 Hybrids of Fullerene and Transition‐Metal Disulfides

As another type of emerging inorganic 2D semiconducting nanomaterials, TMDs have been attracting widespread attention due to their intriguing electronic, optical, and mechanical properties. Molybdenum disulfide (MoS_2_) is the most representative TMD, consisting of hexagonal rings with Mo and S atoms alternately located at the hexagon corners. Monolayer MoS_2_ has the direct band gap of ≈1.8 eV and a high in‐plane carrier mobility, enabling broad applications in electro and photocatalysts, photovoltaics and photoelectric devices.^124^, ^125^

Despite the extensive reports on the applications of MoS_2_ in photocatalytic H_2_ production, quite a low photocatalytic activity of MoS_2_ has been achieved even for the single‐layer MoS_2_ since only its edges have the high catalytic activity, whereas its basal plane is inactive.^124^, ^125^ Following the similar concepts established for graphene and g‐C_3_N_4_, hybridizing MoS_2_ with other functional materials including fullerenes is effective in enhancing the photocatalytic H_2_ production activity of MoS_2_.^56^ In 2005, Remškar et al. used a high‐temperature (1030 K) and catalyzed chemical‐transport reaction to synthesize MoS_2_–C_60_ composite crystals.^126^ The crystals featured a layered structure composed of commutative MoS_2_ and C_60_ molecular layers, as characterized by TEM, electron diffraction, XRD, and XPS. The layered crystal composed of alternating MoS_2_ and C_60_ layers resulted in the MoS_2_–C_60_ composite being a potential photovoltaic material with a high quantum yield of photoinduced charge generation.^126^ Blinc et al. later studied the X‐band electron paramagnetic resonance spectra of a MoS_2_–C_60_ composite and observed a strong temperature‐dependent g‐shift and photoconductivity.^127^

**Figure 23:**
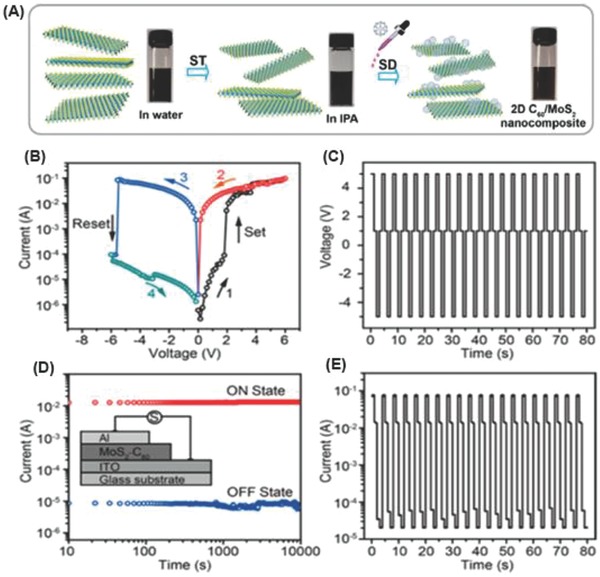
A) Preparation of 2D C_60_–MoS_2_ nanocomposites through solvent transfer (ST) and surface deposition (SD) methods. B) *I–V* characteristics of the ITO/C_60_–MoS_2_/Al diode memory device. C) Retention times of the ON and OFF states of the memory device probed with a voltage of 1.0 V. Inset: Memory device structure. D) Input and E) output of write–read–erase–read (WRER) cycles of the memory device for flash storage applications. Voltages for WRER cycles are +5, 1, −5, and 1 V, respectively. Reproduced with permission.^128^ Copyright 2016, American Chemical Society.

In 2016, Chen, Huang and co‐workers avoided such a harsh condition and synthesized a novel MoS_2_–C_60_ hybrid by the solvent transfer and surface deposition (STSD) method (**Figure** **23**A).^128^ The MoS_2_–C_60_ hybrid showed abundant van der Waals p−n nanoheterojunctions and exhibited extraordinary properties that were not available for the single component of C_60_ or MoS_2_. The authors further investigated the electronic property of the MoS_2_–C_60_ hybrid in a direct charge‐transport diode device configuration of ITO/C_60_−MoS_2_ nanocomposite/Al, which demonstrated a sustainable conductivity bistability and a typical bipolar resistance switching phenomenon with low SET (1.9 V) and RESET (−5.5 V) voltages and a high ON/OFF resistance ratio (≈4 × 10^3^) (Figures 23B–E). A model of the electron tunneling across the junction barriers modulated by electric‐field‐induced polarization was proposed, and the excellent electrical properties of the MoS_2_–C_60_ hybrid originated from the feasibility of the electron tunneling via junction barriers modulated by the electric‐field‐induced polarization.^128^

In 2016, Fang, Banerjee and co‐workers reported a stepwise sulfidation approach for the direct integration of vertically aligned high edge‐density MoS_2_ onto conductive carbon fiber paper (CFP) with exposed catalytically active edge sites, yielding a 3D architecture with a high surface‐to‐volume ratio beneficial to electrocatalytic applications.^129^ Furthermore, the 3D MoS_2_ nanosheets were interfaced with nC_60_ clusters by a facile solution‐deposition method via immersing the MoS_2_/CFP samples into a C_60_ solution in chlorobenzene, and the nC_60_/MoS_2_ hybrid showed greatly improved hydrogen evolution reaction activity with an overpotential value of 172 mV at 10 mA cm^−2^, a Tafel slope of 60 mV dec^−1^, and a turnover frequency value of 2.33 H_2_/s per active site at −0.2 V versus RHE at a C_60_ deposition concentration of 0.5 mg mL^−1^. The improved activity of the nC_60_/MoS_2_ hybrid catalyst was explained by the interfacial doping of MoS_2_ induced by charge transfer from the nC_60_ clusters to MoS_2_, resulting in the relative positioning of their valence and conduction band edges.^129^

In 2018, Imahori et al. fabricated a MoS_2_–C_60_ hybrid onto a semiconducting SnO_2_ electrode via a two‐step methodology.^130^ In the first step, the injection of a poor solvent (acetonitrile, MeCN) into a good solvent NMP with the dispersion of MoS_2_ and C_60_ yielded MoS_2_–C_60_ hybrid aggregates via self‐assembly, followed by electrophoretic deposition onto the SnO_2_ electrode in the second step. The time‐resolved microwave conductivity measurements for the MoS_2_–C_60_ hybrid revealed substantially increased transient conductivities under photoexcitation in comparison with the single components of MoS_2_ and C_60_, indicating that the nanosized heterojunction structure of MoS_2_ and C_60_ promoted charge separation. Furthermore, the C_60_ molecules on the surface of MoS_2_ effectively hindered the undesirable charge combination between an electron in the electrode and a hole in the MoS_2_ nanosheets. Moreover, the authors fabricated a WS_2_–C_60_ hybrid for comparison and found that, despite a more suppressed charge recombination in WS_2_–C_60_ than in MoS_2_–C_60_, the incident photon‐to‐current efficiency value of the device with WS_2_–C_60_ was smaller than that with MoS_2_–C_60_ owing to its inhomogeneous film structure.^130^

To thoroughly understand the basic physical and chemical phenomena that rule highly crystalline architectures comprised of crystals of organic molecules and 2D materials, Wang and co‐workers fulfilled the growth of high‐quality self‐assembled monolayers of C_60_ on WSe_2_, which served as a weakly interacting organic/2D vdW heterostructure system.^131^ The deposited C_60_ molecules formed a monolayer that extended uniformly over WSe_2_ with large grain sizes (≈5 µm). The interplay and balance between the adsorbate–adsorbate and adsorbate–substrate interactions resulted in the formation of rotational arrays of self‐assembled 2 × 2 molecules. According to vdW‐corrected ab initio DFT simulations, the C_60_ molecules tended to be electronically coupled with a long‐range orientational ordering, as reflected in the high crystallinity of C_60_ on WSe_2_. Meanwhile, the electronic structure of the hybrid system showed a spatial delocalization of the molecular orbitals throughout the 2 × 2 superlattice.

Whether the formation of a MoS_2_–fullerene hybrid can promote the photocatalytic H_2_ production activity of MoS_2_ remains intriguing. In 2016, Huang et al. carried out first‐principles calculations based on density functional theory to investigate the effects of vdW interactions on changes in the electronic structure, charge transfer and photoactivity of three typical monolayer MoS_2_/fullerene (C_60_, C_26_, and C_20_) hybrids. For the MoS_2_/fullerene vdW heterostructures, strong noncovalent interactions between the MoS_2_ and fullerene were concluded. The band gap of the MoS_2_/fullerene vdW heterostructure was smaller than that of monolayer MoS_2_, and thus, the visible‐light absorption and photoinduced electron transfer can be enhanced. Furthermore, the amount of charge transfer at interface induced by vdW interactions was found to be dependent on the size of the fullerene.^132^

**Figure 24:**
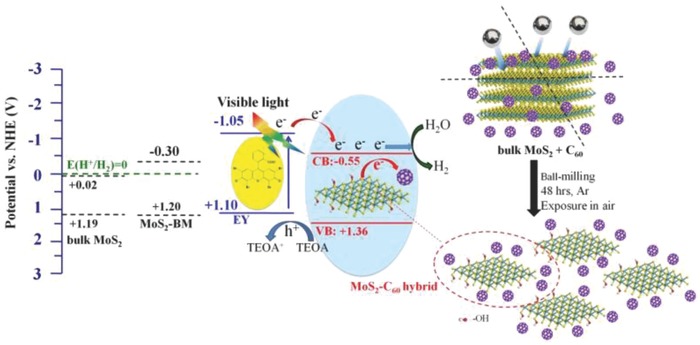
Schematic illustrations of the mechanochemical reaction between MoS_2_ and C_60_ in a sealed ball‐mill crusher (right) and the visible light photocatalytic H_2_ production mechanism of MoS_2_–C_60_ hybrid (left). Reproduced with permission.^67^ Copyright 2018, Elsevier B.V.

Experimentally, our group recently reported the first MoS_2_–C_60_ hybrid featuring a vdW heterostructure, as well as its application in photocatalytic H_2_ production. The MoS_2_–C_60_ hybrid was prepared via a facile and ecofriendly solid‐state mechanochemical route by ball‐milling a mixture of bulk MoS_2_ and C_60_. In the process of ball‐milling, the bulk MoS_2_ was exfoliated to few‐layer MoS_2_ nanosheets, and the basal plane was reduced into significantly smaller nanosheets; while the C_60_ molecules bonded to the edge of the cut MoS_2_ nanosheets in the form of vdW heterostructures (**Figure** **24**).^67^ Upon C_60_ bonding to the edge of the MoS_2_ nanosheets, a negative shift in the conduction band minimum along with a positive shift in the valance band maximum relative to those of bulk MoS_2_ and MoS_2_ ball‐milled without C_60_ (MoS_2_–BM) was revealed (Figure 24). As a result, under the optimized weight ratio of MoS_2_:C_60_ (1:1) in the raw mixture, the MoS_2_–C_60_ hybrid containing 2.8 wt% C_60_ exhibited a visible light photocatalytic H_2_ production rate of 6.89 mmol h^−1^ g^−1^ in the presence of the photosensitizer EY. This value was 9.5 times higher than that of the obtained MoS_2_ nanosheets. This result was because the C_60_ bonding resulted in the fast photoinduced electron transfer from MoS_2_ to C_60_, and this action was beneficial for the separation of electron–hole pairs by inhibiting their recombination.^67^

The photovoltaic applications of C_60_/MoS_2_ and C_60_/WS_2_ hybrids were also studied theoretically by Cheng and co‐workers.^133^ According to the first‐principles calculations on the electronic states and energy level alignments of six ultrathin photovoltaic heterojunctions including C_60_/MoS_2_, C_60_/WS_2_, C_59_B/MoS_2_, C_59_B/WS_2_, C_59_N/MoS_2_, and C_59_N/WS_2_, C_60_ preferred to sit at the S‐top site with a hexagon parallel to the monolayer. All of the interfacial interactions were found to be weak, such that the main electronic features of the components were preserved. Although both the C_60_/MoS_2_ and C_60_/WS_2_ systems behaved as type‐II heterojunctions, the built‐in potential in the C_60_/MoS_2_ hybrid was too small to achieve an efficient electron–hole separation, whereas the latter system had a large built‐in potential and was thus predicted to yield an excellent photovoltaic performance.^133^

## 5 Hybrids of Fullerene and Other 2D Nanomaterials

### 5.1 Hybrids of C_60_ and Hexagonal Boron Nitride

2D h‐BN is a sp^2^‐hybridized wide band‐gap semiconductor with a structure analogous to graphene and belonging to a hexagonal system with sublattices occupied by equal numbers of boron and nitrogen atoms alternatingly arranged in a honeycomb configuration. Similar to graphene, the different h‐BN planes are dominated by weak van der Waals interactions, while the in‐plane nitrogen and boron atoms are combined by sp^2^ orbitals to form strong σ‐bonds, which are highly polarized because of the high asymmetry of the sublattices, resulting in the large band gap (5–6 eV) of h‐BN.^134^ As an indirect band‐gap semiconductor, h‐BN exhibits unique electronic properties with a low dielectric constant, high thermal conductivity, and chemical inertness.^135^ Although h‐BN is electrically insulating, its electronic properties can be facilely modulated by doping, substitution, functionalization, and hybridization.^136^

Given that C_60_ has a narrower band gap, hybridizing h‐BN with C_60_ has been regarded as an effective strategy for modulating the electronic properties of h‐BN. In 2005, Greber and co‐workers grew a C_60_ monolayer film on one monolayer of h‐BN on Ni(111), providing an atomically flat metal‐insulator interface. They observed that, between 150 and 250 K, the work function of C_60_ decreased, while the binding energy of the HOMO increased by ≈100 meV along with a strong charge in the LUMO of C_60_ by 0.4 ± 0.1 electrons. Such a charge redistribution was induced by the onset of a molecular rocking motion, and the magnitude of the charge transfer was an indication of strong electron–phonon interactions.^137^

### 5.2 Hybrid of C_60_ and Black Phosphorus

BP with a century‐long history did not draw significant attention until the recent recognition of its anisotropic 2D layered structure.^138^ The unique band structure, featuring a direct band gap with a thickness‐dependent tunable gap energy in the range of 0.3 to 2.0 eV and a high charge carrier mobility of ≈1000 cm^2^ V^−1^ s^−1^, makes BP an emerging member of 2D nanomaterials and renders its potential for application in transistors, biomedicine, and energy conversion and storage.^139^ BP has a puckered honeycomb structure with an sp^3^‐orbital hybridization, as well as the armchair and zigzag‐configurations along the *x* and *y*‐axial directions, respectively.^140^ Note that, however, 2D BP nanosheets are easily oxidized under ambient condition since each phosphorus atom has a pair of lone electrons that readily react with oxygen adsorbed on the surface of BP nanosheets. Thus, improving the ambient stability of BP is a prerequisite for its practical applications, and hybridizing BP with another stable functional material appears to be a practical solution.^140^, ^141^

Despite extensive reports on hybrids of BP with other 2D nanomaterials, especially graphenes, studies on hybrids of BP and fullerene have rarely been reported. In 2014, Cui and co‐workers made an attempt to synthesize a BP–C_60_ composite via a mechanochemical ball‐milling route in a comparative study with a BP–graphite composite synthesized under the same conditions. Prior to blending with BP, C_60_ or graphite was ball‐milled. Then, the mixture of BP and C_60_ (graphite) was sealed in an argon‐filled glove box, and high‐energy mechanical milling was performed under a 1.2 MPa Ar atmosphere with a rotation rate of 700 rpm for 12 h. According to their detailed characterizations of the BP–graphite composite, phosphorus–carbon bonds formed after ball‐milling and remained stable during lithium insertion/extraction. As a result, an excellent electrical connection between BP and the carbon material was established, contributing to a high initial discharge capacity of 2786 mAh g^−1^ at 0.2 C and an excellent cycle life of 100 cycles with 80% capacity retention. For comparison, within the BP–C_60_ composite, the content of the as‐formed phosphorus–carbon bonds was significantly lower than that for the BP–graphite composite, resulting in an inferior capability. Hence, the authors concluded that the carbon structures played essential roles in the formation of stable phosphorus–carbon bonds.^71^

## 6 Summary and Outlook

The discovery of C_60_ as the first fullerene in 1985 marks a milestone in nanoscience due to its unique spherical molecular structure merely composed of sp^2^‐hybridized carbon atoms. As the only soluble species among all known nanocarbons derived from the 0D structure, fullerenes have been popularly used as important building blocks useful for constructing versatile noncovalent/covalent nanohybrids with a variety of functional materials, including 2D nanomaterials, as elaborately reviewed in this paper.

In 2004, the paradigm‐shifting disclosure of graphene as the first and most representative 2D layered nanomaterial opened up a new era of nanoscience because of the material's exceptionally large specific surface area and high carrier mobility, which are achievable only in the 2D layered structure. The exceptional structure and property of graphene soon stimulated the recognition of other graphene‐analogous 2D nanomaterials, such as g‐C_3_N_4_, TMDs, h‐BN, and BP, which all exhibit similar electronic properties. Meanwhile, their intrinsic differences in lattice composition and interlayer distance bring about a clear diversity in specific functionalities and applications, greatly accelerating the field of 2D nanomaterials. Despite the tremendous progress in investigating the 2D nanomaterials themselves during the past decade, enhancing the existing properties for better performance and even exploring unknown functionalities toward extended applications are definitely necessary. To this end, hybridizing 2D nanomaterials with 0D fullerenes has been well established as a successful and powerful protocol based on the detailed analyses of versatile fullerene‐2D nanomaterial hybrid systems in this Review.

There are yet considerable challenges in the emerging field of fullerene‐2D nanomaterial hybrids. First, most fullerene‐2D nanomaterial hybrids that have been reported so far were synthesized in the noncovalent style via the simple blending of two components, whereas only limited reports involved the successful construction of fullerene‐2D nanomaterial covalent hybrids, which are more promising in terms of strong intermolecular interactions between fullerene and 2D nanomaterial moieties. Clearly, the natural divergence in dimensions between 0D fullerene and 2D nanomaterials makes their covalent connection difficult. Therefore, prior to hybridization, chemically functionalizing the components of fullerene and/or the 2D nanomaterial appears critical. While the chemical derivatization of fullerenes has been well developed, chemical derivatizations of 2D nanomaterials are still in their infancy, and the development of novel derivatization approaches, especially for such less studied 2D nanomaterials as h‐BN and BP, is highly desired.

Another pending problem is how to determine precisely the chemical structures of fullerene‐2D nanomaterial hybrids at the molecular level. In particular, for fullerene‐2D nanomaterial covalent hybrids, the additional pattern of covalent bonding between fullerene and the 2D nanomaterial, i.e., single‐bond addition or cycloaddition onto the fullerene cage, is essential for the disruption of the π‐conjugated system of the fullerene cage. Furthermore, since the properties of 2D nanomaterials are typically anisotropic, the addition site (surface vs edge) where fullerene bonds onto a 2D nanomaterial strongly affects the difficulty of the hybridization and the modulation extent of the 2D nanomaterial within the fullerene‐2D nanomaterial hybrid. For the latter point, the direct visualization of the fullerene addition sites within the hybrid is undoubtedly needed, and thus, further improvement of the resolution of HR‐TEM/scanning tunneling microscope (STM) for clearer morphological characterizations of the geometric structure of the hybrids is of high importance.

As mentioned above, hybridizing 2D nanomaterials with fullerenes may not only fulfill the modulation of the physical/chemical properties of 2D nanomaterials but also induce new properties in the 2D nanomaterials, thus significantly extending the functionalities and applications of 2D nanomaterials. An intriguing question is whether the hybridization strategy can be used to further create unknown functional materials. For instance, on the basis of binary fullerene‐2D nanomaterial hybrids, by involving one or two other functional materials, which are either another 2D nanomaterial or even a 1D nanomaterial (such as a carbon nanotube or boron‐nitride nanotube), ternary or even quaternary hybrids are expected to form. However, due to a lack of experimental reports, whether the properties of such hybrids can surpass their binary counterparts is unknown. With the foreseeable development of chemical derivatizations of 2D nanomaterials, the syntheses of such unknown complex hybrids will likely come true in the near future, making the emerging fullerene‐2D nanomaterial hybrids even more exciting and promising for vast applications.

## Conflict of Interest

The authors declare no conflict of interest.
